# Dose-Effect/Toxicity of Bupleuri Radix on Chronic Unpredictable Mild Stress and Normal Rats Based on Liver Metabolomics

**DOI:** 10.3389/fphar.2021.627451

**Published:** 2021-09-07

**Authors:** Peng Wang, Xiaoxia Gao, Meili Liang, Yuan Fang, Jinping Jia, Junsheng Tian, Zhenyu Li, Xuemei Qin

**Affiliations:** ^1^ Modern Research Center for Traditional Chinese Medicine, Shanxi University, Taiyuan, China; ^2^ Key Laboratory of Chemical Biology and Molecular Engineering of Ministry Education of Shanxi University, Taiyuan, China; ^3^ Scientific Instrument Center, Shanxi University, Taiyuan, China

**Keywords:** bupleuri radix, anti-depression, dose- effect/toxicity, UHPLC-MS, CUMS, liver metabolomics

## Abstract

Depression, one of the most prevalent psychiatric diseases, affects the quality of life of millions of people. Studies have shown that the lower polar fraction of Bupleuri Radix (PBR) elicited therapeutic effects in chronic unpredictable mild stress (CUMS) rats. In contrast, comparatively mild liver injury was observed in normal rats administered a high PBR dose. It is essential to clarify the effective and safe dose of PBR and its dose-effect/toxicity relationship. In this study, we used the CUMS model to evaluate the effects and toxicities of PBR and to decipher the dose-effect/toxicity relationship and mechanism using the liver metabonomics combined with multivariate statistical analysis. In CUMS rats, PBR improved the depression-like behaviors including reduced body growth rate, anhedonia, and locomotor activities, and markedly reduced the contents of alanine aminotransferase (ALT) and aspartate aminotransferase (AST). In control rats, PBR treatment altered ALT and AST from typical levels. Moreover, the effective dose range for CUMS rats was 12.6–163 g (herb)/kg, the median toxicity dose for CUMS and normal rats were 388 and 207 g (herb)/kg. The toxicological results showed that the cytokeratin-18 fragment level was increased significantly in CUMS rats given with 100 g (herb)/kg PBR. After a comprehensive analysis, the use of PBR dose was determined to be 12.6–50 g (herb)/kg. In CUMS rats, PBR could reverse amino acid metabolism, energy metabolism, sphingolipid metabolism, and β-oxidation of fatty acids to produce an anti-depressant effect in a dose-dependent manner. In control rats, two additional metabolic pathways were significantly perturbed by PBR, including glycerophospholipid metabolism and bile acid metabolism. Moreover, the comprehensive metabolic index including dose-effect index (DEI) and dose toxicity index (DTI) had a remarkable ability (ROC = 0.912, ROC = 0.878) to predict effect and toxicity. The DEI and DTI were used to determine the dose range of effect and toxicity which was shown high concordance with previous results. Furthermore, the CUMS rats possessed a higher toxicity tolerance dose of PBR which was consistent with the theory of “You Gu Wu Yun” in traditional Chinese medicine. The metabonomics techniques combined with correlation analysis could be used to discover indicators for comprehensive evaluations of efficacy and toxicity.

## Introduction

Depression, a complicated psychiatric disorder, leads to low morale, weight loss, and anhedonia (Fabricatore and Wadden, 2006; Paykel, 2006). It is considered the fourth leading cause of disability worldwide and the third leading cause of global disease burden (Sayers, 2001; Mathers and Loncar, 2006). The current clinical anti-depressant drugs are ineffective on at least a quarter of patients and produce side effects such as psychomotor impairment, dependence and hepatotoxic reactions (Sarko, 2000; Ishino and Park, 2013; Voican et al., 2013). Traditional Chinese medicines (TCMs) might offer here new options for depression therapy (Liu et al., 2021). Therefore, research studies have paid increasing attention to the TCMs in treating depression.

Bupleuri Radix, the root of *Bupleurum chinense* DC. or *Bupleurum scorzonerifolium* Wild., and is one of the most popular traditional Chinese medicines (TCMs) over the past 2,000 years. The main pharmacological effects of Bupleuri Radix are soothing the liver and relieving depression, evacuating fever, and elevating “Yang Qi” (The Pharmacopoeia Commission of the PRC, 2020). Clinical studies have shown that Bupleuri Radix has also been used for anti-virus infection, anti-acute radiation injury, anti-ulcer effect, reducing blood lipid, inducing serum interferon, enhancing immune function, etc. (Xing, et al., 2017; Jiang et al., 2020). Along with the steadily increasing use of Bupleuri Radix, safety has been highlighted. The effect of “robbing liver yin” of Bupleuri Radix had been widely reported since the Ming and Qing Dynasties (Teo et al., 2016). Modern studies have shown that Bupleuri Radix caused acute liver injury, hepatocyte apoptosis, and acute hepatitis following overdose or long-term, unrestricted and unjustified use (Wang and Song, 2014). Some components in the sibling species of Bupleuri Radix including bupleurotoxin, acetylbupleurotoxin, and saikosaponin D have been reported neurotoxic effect and hepatotoxicity, respectively (Zhang et al., 2014; Zhang F. et al., 2015). In our previous study, the Bupleuri Radix improved depression-like behaviors in chronic unpredictable mild stress rats (CUMS, a depression model of depression, Zhou et al., 2011; Liu et al., 2013). The lower polar fraction of Bupleuri Radix (PBR) had the strongest antidepressant activity than other parts of Bupleuri Radix (Meng et al., 2020). However, comparatively mild liver injury was observed in normal rats administered high doses (Gao et al., 2017).

The liver is the center of material metabolism and energy metabolism. It plays diverse biological roles in oxidative stress and glycogen storage. In traditional Chinese medicine (TCM), the liver has become the main target organ of depression treatment (Jia et al., 2016; Jia et al., 2017). Depression is considered to be “liver qi stagnation”, and relieving “liver qi stagnation” is regarded as an effective method for treating depression in TCM theory (Li, et al., 2020; Chen, et al., 2020a). Xiaoyao San and Radix Bupleuri were the well-known TCM formula for the treatment of depression by relieving “liver qi stagnation” (Liu, et al., 2021; Zhang, et al., 2020). In addition, modern pharmacological studies have shown that chronic stress might cause liver injury by disturbing hepatic function indices (Chen, et al., 2020b), hepatic metabolic profile (Gao, et al., 2017), and the genes expression in phospholipid and primary bile acid biosynthesis pathways (Jia, et al., 2016; Tian, et al., 2017). Furthermore, clinical studies have shown that chronic and acute liver disease patients exhibited different degrees of depression reversely (Nardelli et al., 2013; Suh, et al., 2013; Youssef et al., 2013). Therefore, the liver was selected as a primary target organ of efficacy and toxicity for PBR. Metabonomics is widely used to discover biomarkers and recognize key pathways involved in biological processes (Medina et al., 2014). Currently, Liquid chromatography with mass spectrometry (LC-MS) is becoming the mainstream platform for metabonomics research because of its rapid analysis, high resolution, and high sensitivity (Theodoridis et al., 2012). Advanced analytical techniques of LC-MS and computational methods of metabonomics technologies can provide unique and fundamental insights into disease and therapeutic progression.

**FIGURE 1:**
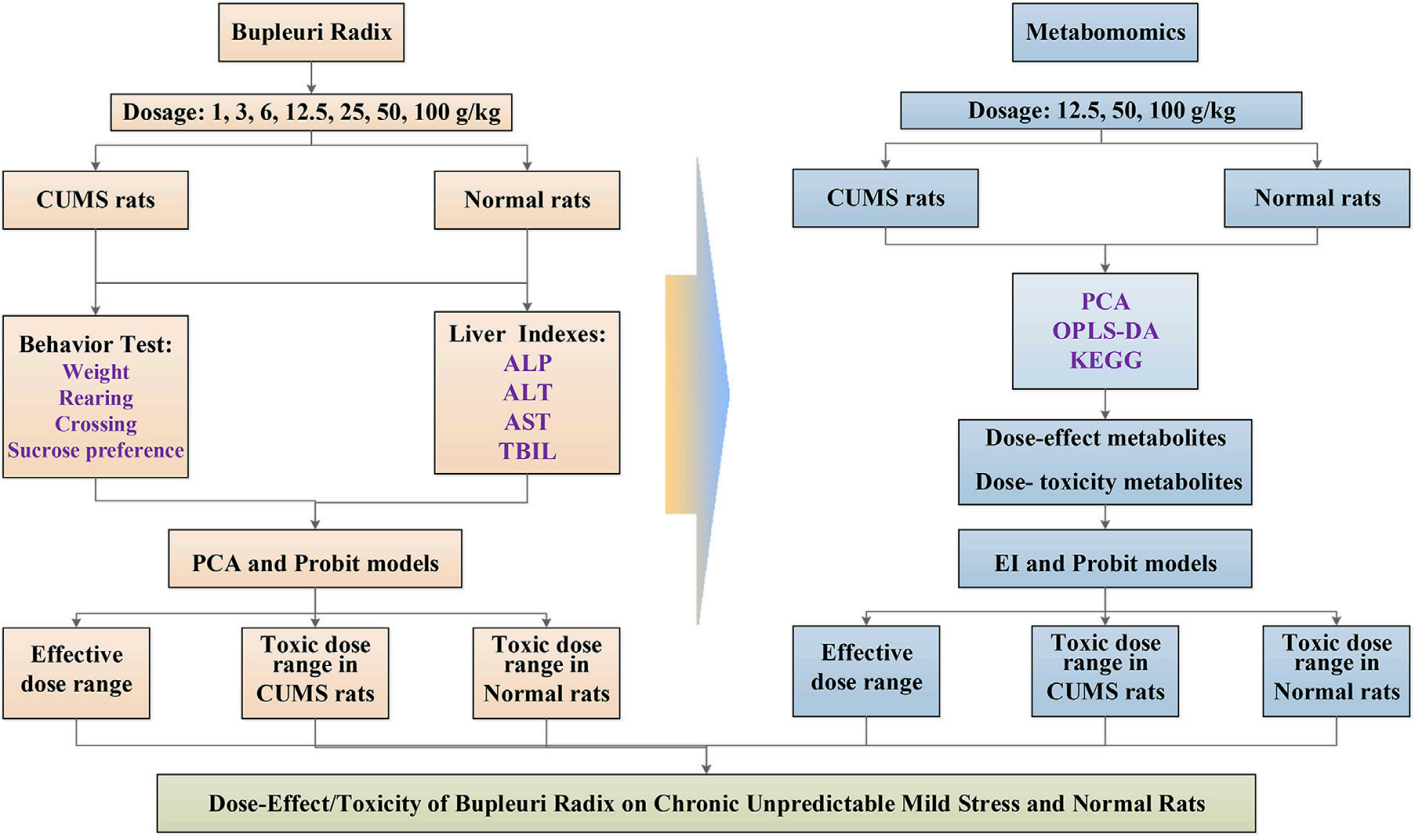
Schematic diagram of dose-effect/toxicity of bupleuri radix on CUMS and normal rats.

In this study, liver metabonomics combined with correlation analysis was applied to characterize the metabolic profile of CUMS rats and the dose-effect/toxicity relationship of PBR in the physiological and pathological conditions of rats (Figure 1). This study provides an objective reference for the evaluation of the safety and effectiveness of PBR.

## Materials and Methods

### Instruments, Chemicals and Reagents

An UltiMate 3,000 ultrahigh performance liquid chromatography (UHPLC, Thermo-Fisher, United States) in tandem with a Q Exactive Orbitrap-MS (Thermo-Fisher Scientific, United States) was used. Xcalibur workstation (Thermo Fisher Scientific, United States), IKA T10 tissue homogenizer (Ningbo Xinzhi Biotechnology Co., Ltd, China), Neofuge13R high-speed freezing centrifuge (Likang Biotechnology Company, China), and MX-S Adjustable mixer (Scilogex, United States) were used. LC-MS grade acetonitrile and HPLC grade formic acid were obtained from Thermo-Fisher (United States).

### Preparation of PBR

The preparation of PBR was briefly described in our previous study (Gao et al., 2017). Five kilograms of Bupleuri Radix (Shanxi Huayang Pharmaceutical Co., Ltd., China) were extracted three times with 95% fresh ethanol, each extraction was performed for 2 h. The mixture was filtered, and the filtrate was concentrated under reduced pressure to obtain an aqueous solution. For further extraction, the aqueous solution was extracted three times with an equal volume of petroleum ether by using 30 min ultrasonication. The petroleum ether fraction (approximately 128.5 g, with an extract yield of 2.57%) was obtained after concentration and drying (60°C).

To assure the quality of PBR, eight polyacetylene compounds from PBR were detected by UPLC Photodiode Array (PDA) analysis in our previous study (Zhang et al., 2017). In this study, the contents of the main active compounds of (2Z, 8E, 10E)-pentadecatriene-4,6-diyn-1-ol and bupleurynol in PBR were 3.62 mg/g and 1.88 mg/g detected by UPLC-PDA. Polyacetylenes accounted for approximately 30% of the total components in PBR, using a quantitative analysis of multiple components by a single marker (QAMS, Xie et al., 2010; Supplementary Figure S1, 2).

PBR extract and venlafaxine was dissolved in distilled water containing 0.5% CMC-Na and 0.5% Tween-80 at seven concentrations of 0.1, 0.3, 0.6, 1.25, 2.5, 5.0, and 10.0 g (herb)/mL for PBR and 3.5 mg/ml for venlafaxine for administration of animal experiments. Among them, the 12.5 g (herb)/kg PBR was regarded as the medium dose, which was based on the effective dose reported in previous research (Gao et al., 2013).

### Animals

136 healthy, adult, 6 weeks old male Sprague-Dawley (SD) rats, 200 ± 20 g weight, license NO. SCXK (JING 2012–0001) were purchased from the Experimental Animal Center of Beijing Weitong Lihua Technology Co. Ltd. (China). The rats were acclimatized for 1 week and maintained on a 12 h light/dark cycle (lights on from 6:00 a.m to 6:00 p.m.), 30–70% relative humidity and temperature (23–27°C) with a commercial diet and water available. All animal experiments were performed under the NIH Guidelines for Care and Use of Laboratory Animals (United States) and the Prevention of Cruelty to Animals Act (1986) of China, and the experiments were approved by the Animal Ethics Committee of Shanxi University (ethical batch number: SXULL2019004).

### Drug Administration and Experimental Design

After 1 week of adaptation, the animals were randomly divided into 17 groups (n = 8): [CM] CUMS rats, [K] control rats, [C1] ∼ [C7] CUMS rats given PBR (7 different dosages), [Z1] ∼ [Z7] control rats administered PBR, and [CY] CUMS rats administered venlafaxine hydrochloride as a positive control (Table1). The different oral doses of PBR were 1, 3, 6, 12.5, 25, 50, and 100 g (herb)/kg, and the oral dose of venlafaxine was 35 mg/kg. All rats were administered agents by gavage at a dose of 10 ml/kg body weight once daily for 21 days, 1 h before modeling.

**TABLE 1:** Animal grouping and processing.

Group	Animal model	Drug	Dosage (g/kg)	Administration
K	—	Solvent	—	Intragastrical
CM	CUMS	Solvent	—	Intragastrical
CY	CUMS	Venlafaxine hydrochloride	0.035	Intragastrical
C1	CUMS	PBR	1	Intragastrical
C2	CUMS	PBR	3	Intragastrical
C3	CUMS	PBR	6	Intragastrical
C4	CUMS	PBR	12.5	Intragastrical
C5	CUMS	PBR	25	Intragastrical
C6	CUMS	PBR	50	Intragastrical
C7	CUMS	PBR	100	Intragastrical
Z1	—	PBR	1	Intragastrical
Z2	—	PBR	3	Intragastrical
Z3	—	PBR	6	Intragastrical
Z4	—	PBR	12.5	Intragastrical
Z5	—	PBR	25	Intragastrical
Z6	—	PBR	50	Intragastrical
Z7	—	PBR	100	Intragastrical

Note: [CM] CUMS rats, [K] control rats, [C1] ∼ [C7] CUMS rats given PBR (7 different dosages), [Z1] ∼ [Z7] control rats administered PBR, and [CY] CUMS rats administered venlafaxine hydrochloride. The solvent was water containing 0.5% CMC-Na and 0.5% Tween-80. All the rats were given agents at a dose of 10 ml/kg body weight.

The control rats were housed together, while the CUMS rats were housed alone and exposed to nine mild stressors randomly every day including swimming in 4°C cold water for 5 min, foot shocking for 2 min (36 V, one shock/2 s, 10 s duration), tail clamping for 2 min, noise for 3 h (60 dB), day-night reversal (12 h/12 h), exposure to an experimental room at 45°C for 10 min, constraint for 3 h, food deprivation for 24 h, and water deprivation for 24 h (Supplementary Table S1; Gao et al., 2017).

### Behavioral Tests and Measurement of Liver Function

During the experiment, all the behavioral tests were measured in day 0 (served as the baseline) and day 21. The rats were weighed and this index reflected the basic survival state of the rats. The open-field test was conducted to measure the number of rearings (defined as standing upright on the hind legs) and the number of crossings (grid lines crossed by the rat with at least three paws). The different forms of activity were used to assess the rats’ mobility. Sucrose preference values were calculated as the percentage of 1% sucrose solution consumed relative to the total liquid intake within 4 h. This test was used to assess anhedonia-like behavior. The growth rate (∆%) of body weight, rearings, crossings, and sucrose preference values were calculated using the formula (F_21_−F_0_)/F_0_, where F_21_ were the body weight, rearings, crossings, and sucrose preference values on the day 21, and F_0_ were the body weight, rearings, crossings, and sucrose preference values on the day 0. The growth rate was used to reflect the behavioral changes in CUMS and control rats between day 0 and 21.

The rats were anesthetized with 10% chloral hydrate at 8:00 a.m. on day 21. Blood was collected from rats 1 h after administration from the abdominal aorta. After 30 min incubation, the serum was separated by centrifugation at 3,500 rpm for 15 min, and then stored at −80°C. Then, the rats were sacrificed by cervical dislocation, and the liver was quickly removed, frozen in liquid nitrogen, and stored at −80°C.

A portion of the serum was used for quantification of alanine aminotransferase (ALT), aspartate aminotransferase (AST), alkaline phosphatase (ALP), and total bilirubin (TBIL) using an automated biochemical analyzer (Konelab PRIME 7.2.1, Finland). Cytokeratin-18 fragment (CK-18F) was quantitatively measured in the serum and liver using the M30 Apoptosense ELISA kit (Shanghai Enzyme-linked Biotechnology Co., Ltd., China). These biochemical indices were examined to assess hepatic function.

### Sample Preparation

To extract metabolites, each liver tissue sample (250 mg) was thawed, and 1,500 μL of chilled acetonitrile containing 0.2% formic acid was added. The sample was homogenized in an ice bath and centrifuged at 13,000 rpm for 15 min at 4°C. The supernatant was transferred to a fresh tube and dried in a refrigerated vacuum centrifugal dryer. The dried residue was dissolved in 500 μL of the solvent containing 0.1% formic acid water-acetonitrile (9:1, *v*/*v*) and centrifuged at 13,000 rpm for 15 min at 4°C. A 5 μL supernatant was injected for UHPLC-MS analysis.

### LC-MS Analysis

UHPLC Q Exactive Orbitrap-MS conditions for liver analysis were the same as those described in our previous study (Gao et al., 2017). UHPLC was performed using a Thermo-Fisher UHPLC coupled to a Q Exactive Orbitrap-MS (Thermo-Fisher, United States). Chromatographic separation was achieved on a Waters ACQUITY UPLC HSS T3 column (2.1 × 100 mm, 1.8 μm) maintained at 40°C. The mobile phase consisted of 0.1% formic acid in water (A) and 0.1% formic acid in acetonitrile (B), respectively, and operated under the following program with a flow rate of 0.2 ml/min: 0–2 min, 2% B; 2–3 min, 2–35% B; 3–17 min, 35–70% B, 17–18 min, 70% B; 18–29 min, 70–98% B; 29–31 min, 98% B; 31–33 min, 98–2% B; 33–35 min, 2% B. The sample injection volume was 5 µL. The mass spectrometer was fitted with an electrospray ionization source, and the ESI source was used in both positive and negative ion modes at a temperature of 320°C. Nitrogen was used as the sheath and auxiliary gas at flow rates of 35 and 10 arb. The heater gas temperature was 300°C, and MS data were collected in the full-scan mode in the m/z range of 100–1,500.

Quality control (QC) samples were processed in the same way as the analytical samples and injected throughout the run to monitor the LC/MS platform’s stability.

### Behavioral Regulation Index (BRI) and Liver Composite Index (LCI)

The behavioral findings and liver biochemical indexes were regarded as the primary evaluation metrics to determine the effect and toxicity dose range of PBR. The behavioral regulation index (BRI) and liver composite index (LCI) were presented as composite scores by combining behavioral indexes and liver biochemical indexes. In this study, principal component analysis (PCA) was used to calculate BRI and LCI, with the formula:

$$f_{1}=k_{1}zx_{1}+k_{2}zx_{2}+k_{3}zx_{3}+k_{4}zx_{4}$$ (1)

$$f_{2}=k_{5}zx_{1}+k_{6}zx_{2}+k_{7}zx_{3}+k_{8}zx_{4}$$ (2)

$$f=\frac{{\text{λ}}_{1}}{{\text{λ}}_{1}+{\text{λ}}_{2}}f_{1}+\frac{{\text{λ}}_{2}}{{\text{λ}}_{1}+{\text{λ}}_{2}}f_{2}$$ (3)

Where *f* was the composite score of BRI or LCI*, f*1 and *f*2 were the first principal component PC1 and the second principal component PC2 of BRI or LCI. *x*_1_, *x*_2_, *x*_3_, and *x*_4_ represented body weight, sucrose preference, ambulation number, and rearing number for BRI or the levels of ALT, AST, ALP, and TBIL for LCI, zx was the standardized raw variable of *x*_i_, λ was the rate of variance contribution, and *k* was the score of each group for every principal component.

The determination of the effective dose range for CUMS rats and the TD50 (median toxicity dose) for CUMS and control rats was as follows. Firstly, the effective and safe range was determined respectively based on the behavior and liver function of control rats (Mean ± 1.96SD, Xie et al., 2010). Secondly, after administration with PBR, the CUMS rats adjusted to the effective range were defined as positive samples for effect, and the CUMS and control rats deviated from the safe range were defined as positive samples for toxicity. Finally, the numbers of positive samples were counted in different groups, and probit regression was used to determine the dose range of effect and toxicity.

### Data Processing and Statistical Analysis

Raw data were processed using Compound Discoverer 2.0 (Thermo Fisher, United States) to obtain the matched and aligned peak data. The processing parameters were as follows: m/z 100–1,500 Da; mass tolerance 5 ppm, RT tolerance 0.05 min, and S/N threshold 3.

The data matrix was established by aligning peaks with the exact retention time and normalizing the peak value. The (Rt)-m/z pair from each file was subjected to multivariate analysis using SIMCA-P software (version 13.0, Umetrics, Sweden), including PCA, partial least-squares discriminant analysis (PLS-DA), and orthogonal partial least squares (OPLS) discriminant analysis of the data from both positive and negative modes. PCA was performed to discern the natural separation between different stages of the samples by visual inspection of the score plots. The PLS-DA model was acquired by projecting the predicted variables and observable variables into a new space. In the OPLS-DA model, samples from different groups were classified, and the results were visualized as score plots to show the group clusters, and S-plots to show the variables contributing to classification. The variable importance in the projection (VIP) value reflects the influence of every variable on the classification, and the independent sample *t*-test was also included in the analysis of the discriminating variables. *p* < 0.05, and VIP>1 were considered statistically significant.

The Human Metabolome Database (HMDB), KEGG, and the LIPI\MAPS-Nature Lipidomics Gateway (http://www.lipidmaps.org/) and related literature were queried with the exact masses of the metabolites to identify the differential metabolites and to understand better the metabolic pathways affected by CUMS stress or PBR. The metabolite correlation network and metabolic networks were constructed using Cytoscape software (v.3.5.0).

### Dose-Effect and Dose-Toxicity Metabolic Screening

For further screening of the dose-effect and dose-toxicity metabolites, the related metabolites were analyzed by correlation analysis (SPSS 21, Pearson correlation analysis) between the different metabolites and the different dosages with *p* > 0.05. The dose-effect and dose-toxicity metabolites were obtained based on regression analysis (Prism 8.0, linear regression analysis) between the related metabolites and dosages with R > 0.6.

### Construction and Evaluation of Dose-effect and Dose-toxicity Models

In this study, four models were used to integrate dose-response and dose-toxicity metabolites, including PCA, effect index (EI), SUM, and (R) SUM (additivity related to metabolites’ R-values), and then the ROC curve was used to evaluate the performance of the four models and metabolites.

### Dose-Effect and Dose-toxicity Index Calculations and Evaluations

In this study, the dose-effect index (DEI) and dose toxicity index (DTI) of each sample was used as the composite indicator. The formula for calculating DEI and DTI was as follows (Zhang ZZ. et al., 2015).

$$DEI=\overset{n}{\underset{i=1}{\sum }}\left|\frac{Ci-\bar{CMi}}{\bar{CMi}-\bar{Ki}}\right|\times 100\%$$ (5)

$$DTI=\overset{n}{\underset{i=1}{\sum }}\left|\frac{Zi-\bar{Ki}}{\bar{Ki}}\right|\times 100\%$$ (6)

Ci was the relative level of one of the metabolites in every rat in the C4, C6, and C7 groups, Zi was the relative level of one of the metabolites in each rat in the Z4, Z6, and Z7 groups, Ki was the average relative level of one of the metabolites in the K group, and $\bar{CMi}$ was the average relative level of one metabolite in the CM group.

The dose-effect and dose-toxicity relationships were evaluated using regression analysis (Prism 8.0, linear regression analysis).

### Statistical Analysis

Quantitative data were presented as the mean ± SD. The significance of differences between groups in terms of behavioral changes was determined using one-way ANOVA with SPSS software (version 21.0). Student’s t-test was used to compare two groups, and statistical significance was set at *p* < 0.05.

## Results

### The Dose Range of the Effects and Toxicity in CUMS and Control Rats Administered PBR

#### Behavioral and Liver Function Indexes on CUMS and Control Rats Administration With PBR

The results of the behavioral tests, including body weight, sucrose preference test, and open-field test during the stress period of 21 days in CUMS and control rats are shown in Table 2 and Supplementary Table S2. PCA was applied to the behavior indexes, and the PCA scores were expressed in two-dimensional scatter plots (PC1 and PC2 plotted on the *x* and *y* axes, respectively). The PCA score plot revealed the separation of CUMS rats from normal rats, suggesting that depressive-like behaviors developed in CUMS rats (Supplementary Figure S3A). The PCA score plot of C1-C7 groups was observed in the K and CM groups, indicating that the depressive-like behavior of CUMS rats was altered by PBR administration but existed in an effective dose range. As shown in Table 2, the growth rate (∆%) of bady weight, rearings, crossings and sucrose preference values slower significantly (*p* < 0.05) in CUMS rats compared with the control group, and the concentration range of 12.5–100 g/kg (C4-C7) of the PBR significantly reversed the decrease of the growth rate, especially in the C6 group (50 g/kg), suggesting that PBR played an anti-depression role in improving the slow body weight gain, anhedonia, and locomotor activities in CUMS rats. However, there was no distinct impact of PBR on control rats (Table 2 and Supplementary Table S2).

**TABLE 2:** Behavior on CUMS and healthy rats administration with PBR (Mean ± SD, n = 8).

Group	Weight (Δ%)	Rearings (Δ%)	Crossings (Δ%)	Sucrose preference value (Δ%)
C1	0.351 ± 0.011**	−0.394 ± 0.181^##^	−0.261 ± 0.037**	−0.37 ± 0.053**^##^
C2	0.311 ± 0.006*	−0.486 ± 0.259^##^	−0.303 ± 0.075*	−0.18 ± 0.045^#^
C3	0.339 ± 0.007**	−0.532 ± 0.106^##^	−0.342 ± 0.187^#^	−0.22 ± 0.064^##^
C4	0.342 ± 0.008**	0.294 ± 0.112*^##^	−0.253 ± 0.102**	−0.293 ± 0.076*^##^
C5	0.308 ± 0.001*	0.283 ± 0.201**^#^	−0.186 ± 0.070**	−0.24 ± 0.034^##^
C6	0.312 ± 0.004*	0.126 ± 0.042**	0.134 ± 0.044**^##^	0.188 ± 0.051*
C7	0.181 ± 0.009*^##^	0.052 ± 0.022**	−0.032 ± 0.008**^##^	−0.29 ± 0.083^#^
Z1	0.312 ± 0.007**	0.094 ± 0.081**	−0.277 ± 0.048*	0.136 ± 0.025*
Z2	0.316 ± 0.005**	0.104 ± 0.059**	−0.268 ± 0.059*	0.166 ± 0.048*
Z3	0.318 ± 0.017**	0.148 ± 0.086**	−0.312 ± 0.086*	0.103 ± 0.053*
Z4	0.314 ± 0.002**	0.078 ± 0.051**	−0.329 ± 0.102*	0.149 ± 0.063*
Z5	0.308 ± 0.007**	0.114 ± 0.014**	−0.316 ± 0.081*	0.114 ± 0.074*
Z6	0.315 ± 0.004**	0.126 ± 0.053**	−0.283 ± 0.039*	0.124 ± 0.048*
Z7	0.309 ± 0.011**	0.058 ± 0.036**	−0.296 ± 0.019*	0.096 ± 0.043*
CM	0.243 ± 0.008^##^	−0.381 ± 0.235^##^	−0.3932 ± 0.193^##^	−0.2 ± 0.028^#^
CY	0.294 ± 0.014*	−0.398 ± 0.179^##^	−0.292 ± 0.075*	0.118 ± 0.022*
K	0.313 ± 0.009**	0.121 ± 0.083**	−0.307 ± 0.096*	0.037 ± 0.009*

Note: [CM] CUMS rats, [K] control rats, [C1] ∼ [C7] CUMS rats given PBR (7 different dosages), [Z1] ∼ [Z7] control rats administered PBR, and [CY] CUMS rats administered venlafaxine hydrochloride. #, compared with the K group (#*p* < 0.05, ##*p* < 0.01); *, compared with the CM group (*p < 0.05, **p < 0.01).

Similarly, PCA was applied for liver function indices, and no obvious distinction was observed in the PCA score plot of each group (Supplementary Figure S3B), suggesting that there were no significant differences in liver function between the groups. However, deviations were observed in the C1-C7, Z1-Z7, and CM groups compared with the normal group, occurring at various levels, suggesting that the modeling and administration caused changes in body liver function. As shown in Table 3, the levels of ALT and AST were markedly reduced in CUMS rats administered with low-dose and medium-dose treatment with PBR (C1-C5, 1–25 g/kg), suggesting that the lower doses of PBR showed obvious protective effects on liver function. While in control rats (Table 3), high-dose and medium-dose-treatment of PBR (Z4-Z7, 12.5–100 g/kg) deviated ALT and AST from the normal range, suggested that PBR in the concentration of D4 ∼ D7 could cause liver injury in control rats. Thus, further research could be carried out to explore whether the administration of PBR caused liver damage and the toxicity dose range of PBR in CUMS and normal rats.

**TABLE 3:** Liver function indexes in CUMS and healthy rats administration with PBR (Mean ± SD, n = 8).

Group	ALP	ALT	AST	TBIL
C1	226.5 ± 36.7^##**^	51.8 ± 6.3^**^	129.3 ± 8.6^**^	11.1 ± 2.6
C2	196.6 ± 39.7	59.1 ± 21.6^*^	121.5 ± 21.1^#***^	7.3 ± 1.0^#^
C3	193.9 ± 28.4	64.1 ± 37.9	120.6 ± 10.2^#**^	5.5 ± 2.7^#^
C4	176.8 ± 38.6	62.0 ± 9.3	118.3 ± 26.8^#***^	6.7 ± 2.9^#^
C5	229.5 ± 45.1	59.5 ± 13.4	137.2 ± 30.5^*^	7.7 ± 1.5^#^
C6	267.8 ± 46.36^##**^	67.1 ± 11.4	154.3 ± 18.6	9.2 ± 3.3
C7	251.6 ± 50.3^###***^	72.8 ± 14.9	141.1 ± 32.7^*^	7.7 ± 1.5^#^
Z1	189.9 ± 28.6	64.2 ± 8.5	141.7 ± 17.6	9.6 ± 1.9
Z2	196.2 ± 28.6	66.9 ± 11.7	149.6 ± 15.5	10.7 ± 2.1^*^
Z3	235.3 ± 65.2^##^	69.5 ± 11.4	161.7 ± 29.3	13.3 ± 5.2^*^
Z4	186.9 ± 22.7	84.7 ± 20.6	152.6 ± 22.0	10.3 ± 4.3^*^
Z5	200.7 ± 37.0^##^	87.6 ± 14.1	179.6 ± 25.2	10.0 ± 3.3^*^
Z6	240.2 ± 55.1^##^	69.3 ± 12.1	123.5 ± 28.3^##^	9.0 ± 4.0^*^
Z7	251.6 ± 50.3^###^	68.6 ± 19.4	148.9 ± 35.6	6.8 ± 1.2^#^
CM	169.9 ± 24.4	78.8 ± 26.2	172.8 ± 44.6	8.7 ± 3.5^#^
K	173.4 ± 19.4	64.0 ± 17.0	156.8 ± 44.5	10.8 ± 2.3^*^

Note: [CM] CUMS rats, [K] control rats, [C1] ∼ [C7] CUMS rats given PBR (7 different dosages), [Z1] ∼ [Z7] control rats administered PBR, and [CY] CUMS rats administered venlafaxine hydrochloride. #, compared with the K group (#*p* < 0.05, ##*p* < 0.01, ###*p* < 0.001); *, compared with the CM group (*p < 0.05, **p < 0.01, ***p < 0.001).

#### PCA Analysis of BRI and LCI

For BRI, the variance contribution rate of the first principal component PC1 was 53%, and PC2 was 28%, accounting for 81% of the information of the original data set cumulatively (>80%). For LCI, the variance contribution rates of PC1 and PC2 were 52.9 and 35.5%, respectively, and the total variance contribution rate was 89% (>80%). The BRI and LCI can be expressed as Eqs 6–8 and Eqs 9–11.

For BRI,

$$f_{1}=0.517zx_{1}-0.131zx_{2}+0.52zx_{3}+0.165zx_{4}$$ (6)

$$f_{2}=0.196zx_{1}+0.66zx_{2}+0.169zx_{3}-0.623zx_{4}$$ (7)

$$f_{\left(BRI\right)}=0.65f_{1}+0.35f_{2}$$ (8)

For LCI,

$$f_{1}=0.115zx_{1}+0.758zx_{2}+0.803zx_{3}+0.721zx_{4}$$ (9)

$$f_{2}=0.977zx_{1}+0.134zx_{2}-0.212zx_{3}-0.061zx_{4}$$ (10)

$$f_{\left(LCI\right)}=0.6f_{1}+0.4f_{2}$$ (11)

#### The Dose Range of Effect and Toxicity Based on Behavior and Liver Indexes

From these two standard accuracy indices of the K group, the threshold values of efficacy and toxicity were determined (mean±1.96 SD) and used to screen for the effects and toxicity responses (Table 4). Ultimately, the probit function was used to calculate the dose range. The obtained effective dose range for the CUMS rats was from 12.6 g/kg to 163 g/kg (EC_50_–EC_95_) with the regression function PROBIT(*P*) = –0.139 + 0.11x, and the TC_50_ for CUMS and normal rats was 388 g/kg and 207 g/kg with the regression function PROBIT(*P*) = –1.156 + 0.03xand PROBIT(*P*) = –1.669 + 0.081x. The results suggested that a broad effective dose range of PBR was observed in CUMS rats, and the lowest dose (12.6 g/kg) was consistent with these results in the behavioral test. The subthreshold toxicity doses of the CM group were higher than those of normal rats administered PBR, which revealed that the liver function of normal rats was more susceptible to PBR. However, the specific mechanism of effect and toxicity of PBR in CUMS and normal rats remained to be determined.

**TABLE 4:** The rates of effect and toxicity on CUMS and normal rats administration with PBR.

Dose (g/kg)	Total samples	Effect [BRI, efficiency rate (%)]		Toxicity [LCI, toxicity rate (%)]	
		CUMS rats	Control rats	CUMS rats	Control rats
0	8	0	100	25	0
1	8	50	100	0	0
3	8	25	100	12.5	12.5
6	8	75	100	12.5	0
12.5	8	62.5	100	12.5	12.5
25	8	62.5	100	25	0
50	8	75	100	0	25
100	8	75	100	25	12.5

Comprehensive indicators, including BRI and LCI, showed a dose-dependent effect, and the regression models were used for dose-effect/toxicity relationship assessment (Supplementary Figure S4). The results showed that BRI and LCI exhibited a strong dose-effect/toxicity relationship evaluation (R > 0.8).

#### CK-18F Levels in Serum and Liver Samples of CUMS and Control Rats Administered PBR

**FIGURE 2:**
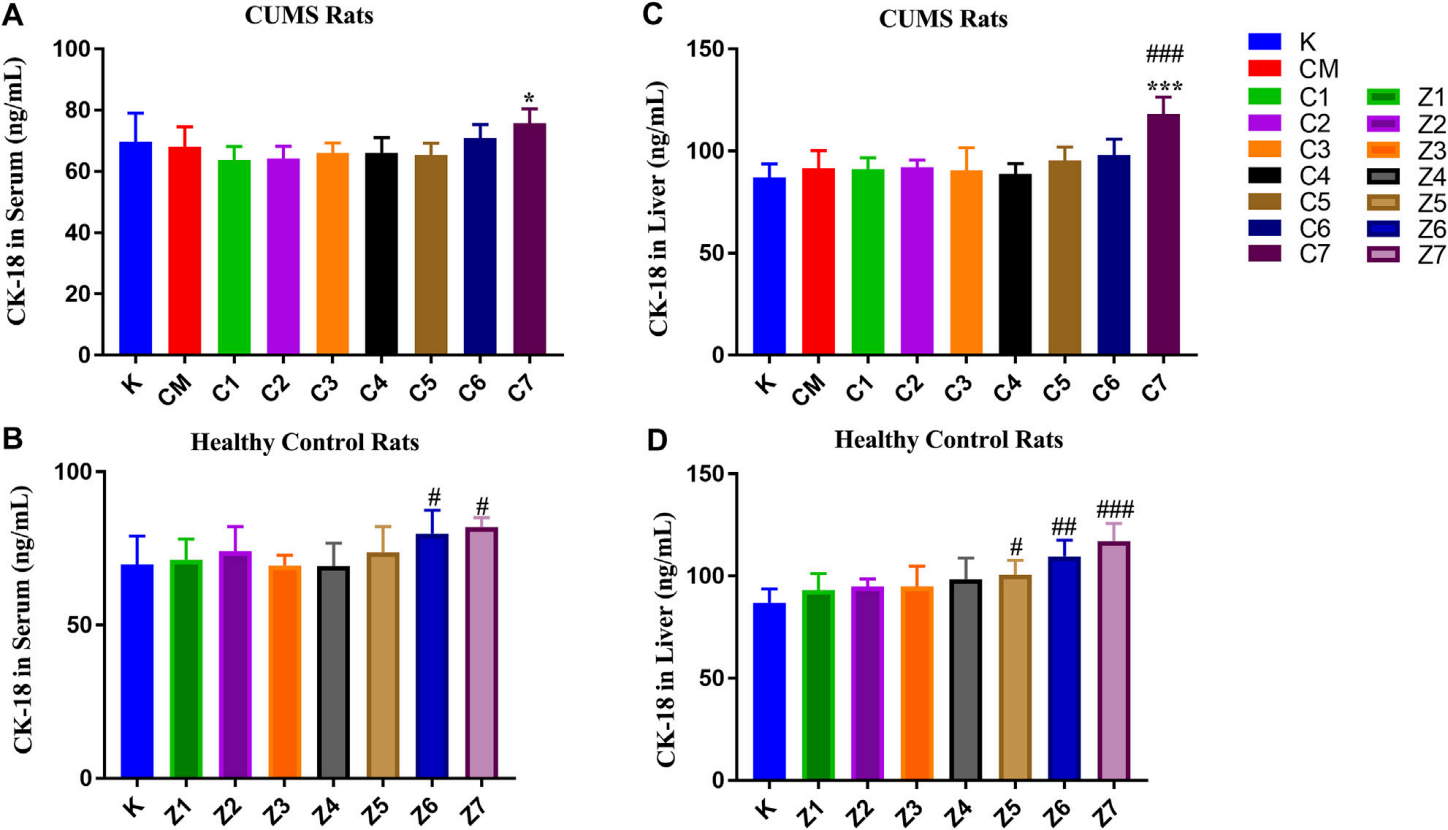
CK-18F level in serum samples of CUMS rats **(A)** and control rats **(B)** administration with PBR. CK-18F level in liver samples of CUMS rats **(C)** and control rats **(D)** administration with PBR. [CM] CUMS rats, [K] control rats, [C1] ∼ [C7] CUMS rats given PBR (7 different dosages), [Z1] ∼ [Z7] control rats administered PBR, and [CY] CUMS rats administered venlafaxine hydrochloride. Data represent the mean ± SD of each group. #, compared with the K group (#*p* < 0.05, ##*p* < 0.01, ###*p* < 0.001); *, compared with the CM group (*p < 0.05, ***p < 0.001).

As shown in Figure 2, there was no significant difference in the CK-18F level in serum and liver between the CM and K groups. In CUMS rats, the content of CK-18F did not significantly change in the serum of C1-C7 groups (Figure 2A) but increased significantly in the liver of the C7 group compared with the K group (*p* < 0.001, Figure 2B), suggesting that rats administrated with 100 g/kg PBR exhibited severe liver damage. After a comprehensive analysis, the effective dose range of PBR was 12.6–50 g/kg. In normal rats, CK-18F levels in the serum of the Z6-Z7 group were significantly increased (*p* < 0.05) (Figure 2C). In the liver, the CK-18F level in the Z5-Z6 groups was increased (*p* < 0.05) and significantly increased in the Z7 group compared with the K group (*p* < 0.001, Figure 2D).

To determine the specific mechanism of effect and toxicity of PBR in CUMS and normal rats, the lowest effective dose [C4] group, the most effective dose [C6] group, and the toxic dose [C7] group, and three corresponding groups [Z4], [Z6], and [Z7] were chosen for metabolomics analysis.

### PBR Adjusted Differentially on Metabolites of Rat Liver by UHPLC-MS

#### Assessment of Stability in UPLC-MS System

**FIGURE 3:**
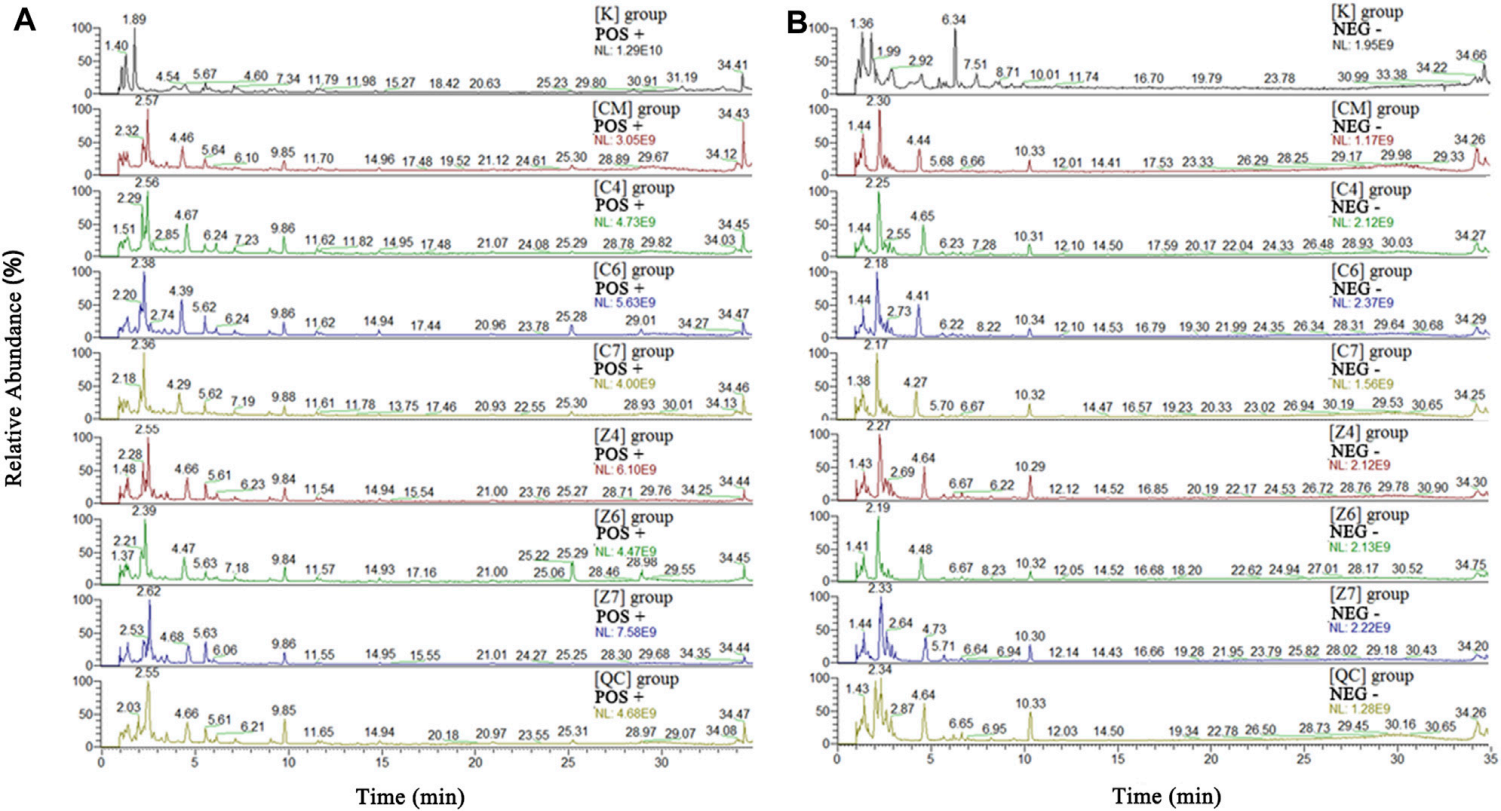
Total ions chromatograph (TIC) in positive **(A)** and negative **(B)** ion of [K], [CM], [C1]–[C7], [Z1]–[Z7]and [QC] group. [CM] CUMS rats, [K] control rats, [C1] ∼ [C7] CUMS rats given PBR (7 different dosages), [Z1] ∼ [Z7] control rats administered PBR, and [CY] CUMS rats administered venlafaxine hydrochloride.

The metabolic profiles of the liver samples were characterized using UHPLC-Q Exactive Orbitrap-MS. Typical total ions chromatograph (TIC) of [CM], [C4], [C6], [C7], [K], [Z4], [Z6], and [Z7] showed approximately 8,671 ions, and 409 metabolites were identified in the sample (Figure 3). The metabolites were mainly high polarity compounds including amino acids, sphingolipids, bile acids, etc.

The stability of the LC-MS system was assessed using QC samples. The PCA score plot indicated that the QC samples were tightly clustered (Supplementary Figure S5). Moreover, the peak areas and retention times of the ten extracted ions in the QC samples also showed good stability (Supplementary Table S3). The RSDs of the ten peaks were 0.22–3.85% for retention times, 3.76–9.66 × 10^–5^% for the *m*/*z* value, respectively. These results indicate that the UPLC-MS system is robust for metabolomic analysis.

#### Multivariate Statistical Analysis

**FIGURE 4:**
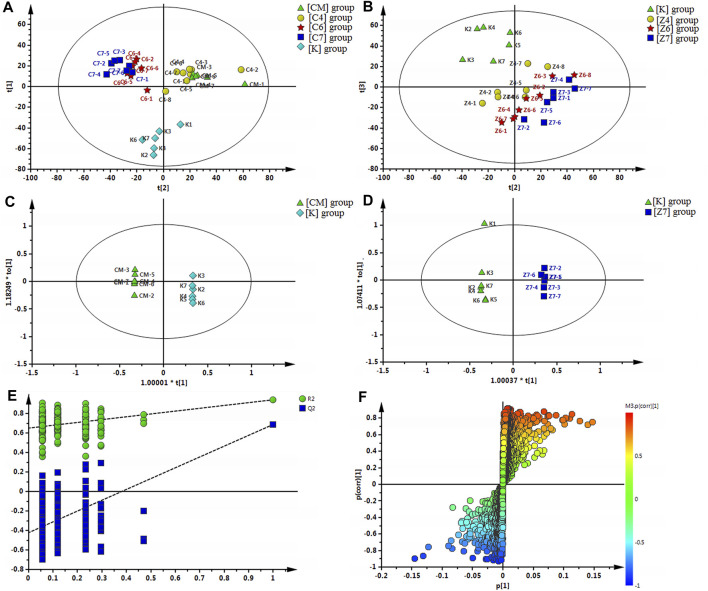
**(A)** Multiple pattern recognition of liver metabolites in CUMS rats with or without exposure to PBR. PLS-DA score plot (n = 8, R2Y = 0.901, R2X = 0.51, Q2 = 0.651). **(B)** PCA of liver metabolites of normal rats with or without exposure to PBR. PCA score plot (n = 4, R2X = 0.531, Q2 = 0.149). **(C)** OPLS score plotof [K] group and [CM] group (n = 8, R2Y = 1, R2X = 0.455, Q2 = 0.868). **(E)** OPLS S-plot of [K] group and [CM] group. **(D)** OPLS score plot (n = 8, R2Y = 1, R2X = 0.337, Q2 = 0.59) of [Z7] group and [K] group and the corresponding OPLS S-plot **(F)**. [CM] CUMS rats; [K] control rats; [C4], [C6], and [C7] CUMS rats given PBR (12.6, 50, 100 g/kg); [Z1], [Z6] and [Z7] control rats administered PBR (12.6, 50, 100 g/kg).

A snapshot of the systematic metabolism in the [CM], [K], [C4], [C6], and [C7] groups was obtained using PLS-DA pattern recognition analysis. An obvious separation was observed between [CM] and [K], indicating that the alterations in metabolic profiles were related to CUMS stress. In addition, [C4], [C6], and [C7] moved away from [CM] in a dose-dependent manner in PLS-DA (Figure 4A), suggesting that the differences in metabolic profiles were related to the effects of PBR on CUMS-induced rats. The reliability of the multiple pattern recognition methods was evaluated by the R^2^Y and Q^2^ values (R^2^Y represents goodness of fit, and Q^2^ indicates goodness of prediction). The R^2^Y and Q^2^ were 0.901 and 0.651, indicating that the PLS-DA model could accurately describe the data. These results of the permutation tests showed that the two models were credible without overfitting (Figure 4A).

#### CUMS Induced Deviation of Liver Metabolic Profile in Rats

**FIGURE 5:**
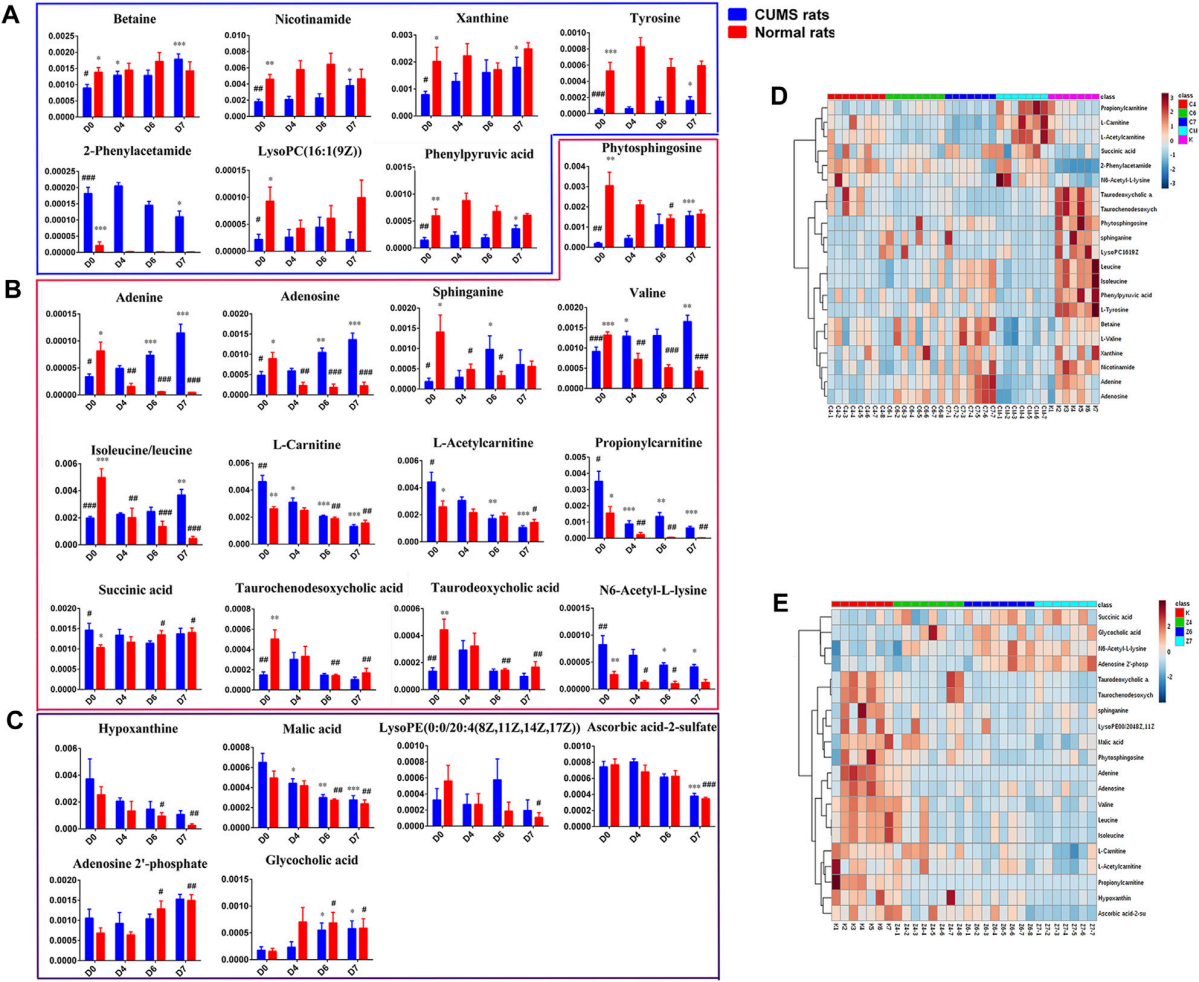
**(A)**: Differential metabolites only in liver samples of CUMS rats with PBR treatment. **(B)**: Common differential metabolites in liver samples of both CUMS and normal rats with PBR administration. **(C)** Differential metabolites only in liver samples of normal rats with PBR administration. **(D)**: Relative content of the heatmap of differential metabolites in liver samples of CUMS rats with PBR treatment. **(E)**: Relative content of the heatmap of differential metabolites in liver samples of normal rats with PBR administration. (Normalized intensity levels of differential metabolites in liver samples of CUMS rats (■blue), and the relative content of the corresponding metabolites in normal rats (■red)). D0: administration of an equal volume of vehicle. D4-D7: the concentration of PBR was 12.5, 5, and 10 g/kg, respectively. (Expressed as the volume of raw materials). Data are presented as mean ± SEM. n = 8 each group. *p < 0.05, **p < 0.01, ***p < 0.001 vs [CM] group; #*p* < 0.05, ##*p* < 0.01, ###*p* < 0.001 vs. [K] group; [CM] CUMS rats; [K] control rats; [C4], [C6], and [C7] CUMS rats given PBR (12.6, 50, 100 g/kg); [Z1], [Z6] and [Z7] control rats administered PBR (12.6, 50, 100 g/kg). The ribbon −4∼4: represents the relative content of the differential metabolites from low to high.

To discover the liver metabolites between the [CM] and [K] groups, OPLS-DA analysis was applied to eliminate unrelated variations in the spectra. The OPLS-DA score plot showed a statistically significant difference between the [CM] and [K] groups (Figure 4C), indicating a similar result that metabolic profiles were significantly altered in rat liver with CUMS stress. The corresponding OPLS S-plot (Figure 4E) in turn showed the contribution of different variables and ions far away from the origin were deemed as potential biomarkers. As a result, 21 candidates were screened from the corresponding S-plot between the [CM] and [K] groups with VIP > 1.0. The changes in these potential biomarkers are listed in Table 5 and Figures 5A,B,D. Compared with the [K] group, the levels of 15 metabolites, including leucine, betaine, isoleucine, valine, phenylpyruvic acid, l-tyrosine, nicotinamide, adenine, adenosine, xanthine, sphinganine, lysoPC (16:1 (9Z)), phytosphingosine, taurodeoxycholic acid, and taurochenodeoxycholic acid, decreased significantly in the [CM] group. In contrast, six metabolites, including 2-phenylacetamide, N6-acetyl-l-lysine, l-carnitine, l-acetylcarnitine, propionylcarnitine, and succinic acid, increased significantly in the [CM] group.

**TABLE 5:** Identified differential metabolites in the liver of CUMS rats.

No	Metabolites	TR (min)	m/z	Ion	CM/K	C7/CM	C6/CM	C4/CM	Metabolic pathway
1	Betaine	1.82	118.0864	M + H	↓^a^	↑^a^	↑	↑^a^	*Glycine*, serine and threonine metabolism
2	Leucine^b^	3.16	132.1025	M + H	↓^a^	↑^a^	↑^a^	↑^a^	Amino acid metabolism
3	Isoleucine^b^	3.42	132.1025	M + H	↓^a^	↑^a^	↑^a^	↑^a^	Amino acid metabolism
4	Valine^b^	1.40	118.0864	M + H	↓^a^	↑^a^	↑	↑^a^	Amino acid metabolism
5	2-Phenylacetamide	3.43	136.0756	M + H	↑^a^	↓^a^			Phenylalanine metabolism
6	Phenylpyruvic acid	3.13	165.0533	M-H	↓^a^	↑^a^	↑	↑	Phenylalanine metabolism
7	l-Tyrosine	2.78	182.0811	M + H	↓^a^	↑^a^	↑	↑	Phenylalanine metabolism
8	N6-Acetyl-l-lysine	3.31	189.1232	M + H	↑^a^	↓	↓^a^	↓^a^	Lysine degradation
9	Nicotinamide	1.85	123.0554	M + H	↓^a^	↑^a^	↑	↑	Nicotinate and nicotinamide metabolism
10	Adenine	3.79	136.062	M + H	↓^a^	↑^a^	↑^a^		Purine metabolism
11	Adenosine	3.46	268.1036	M + H	↓^a^	↑^a^	↑^a^	↑	Purine metabolism
12	Xanthine	2.80	153.0406	M + H	↓^a^	↑^a^	↑	↑	Purine metabolism
13	l-Carnitine	1.39	162.1126	M + H	↑^a^	↓^a^	↓^a^	↓^a^	β-oxidation of fatty acid
14	l-Acetylcarnitine	2.36	204.1229	M + H	↑^a^	↓^a^	↓^a^	↓	β-oxidation of fatty acid
15	Propionylcarnitine	4.45	218.1386	M + H	↑^a^	↓^a^	↓^a^	↓^a^	β-oxidation of fatty acid
16	Sphinganine	14.41	302.3058	M + H	↓^a^	↑	↑^a^	↑	sphingolipid metabolism
17	Phytosphingosine	11.73	318.3004	M + H	↓^a^	↑^a^	↑	↑	sphingolipid metabolism
18	Succinic acid	2.99	117.0185	M-H	↑^a^		↓	↓	Citrate cycle
19	LysoPC [16:1 (9Z)]	15.94	494.3237	M + H	↓^a^		↑		Glycerophospholipid metabolism
20	Taurodeoxycholic acid^b^	8.34	500.3014	M + H	↑^a^	↑			Bile acid metabolism
21	Taurochenodesoxycholic acid	10.27	500.3014	M + H	↓^a^	↑			Bile acid metabolism

Note: [CM] CUMS rats, [K] control rats, [C1] ∼ [C7] CUMS rats given PBR (7 different dosages). ↑ shows up-regulated metabolite; ↓ shows down-regulated metabolite.

a Means a statistically significant difference at *p* < 0.05.

b Validated with standard. [CM] group compared with [K] group, CM/K; [C4] group compared with [CM] group, C4/CM; [C6] group compared with [CM] group, C6/CM; [C7] group compared with [CM] group, C7/CM.

#### PBR Reversed Metabolic Profile in CUMS-induced Rats

After the administration of PBR, 17 differential metabolites were call-backed close to the [K] group after PBR treatment in the [C4], [C6], and [C7] groups, except l-carnitine, l-acetylcarnitine, and propionylcarnitine with an over-reversed regulation at the D7 dose. LysoPC (16:1 (9Z)), taurodeoxycholic acid, taurochenodesoxycholic acid, and succinic acid were not reversed at D6 and D7 of PBR.

**FIGURE 6:**
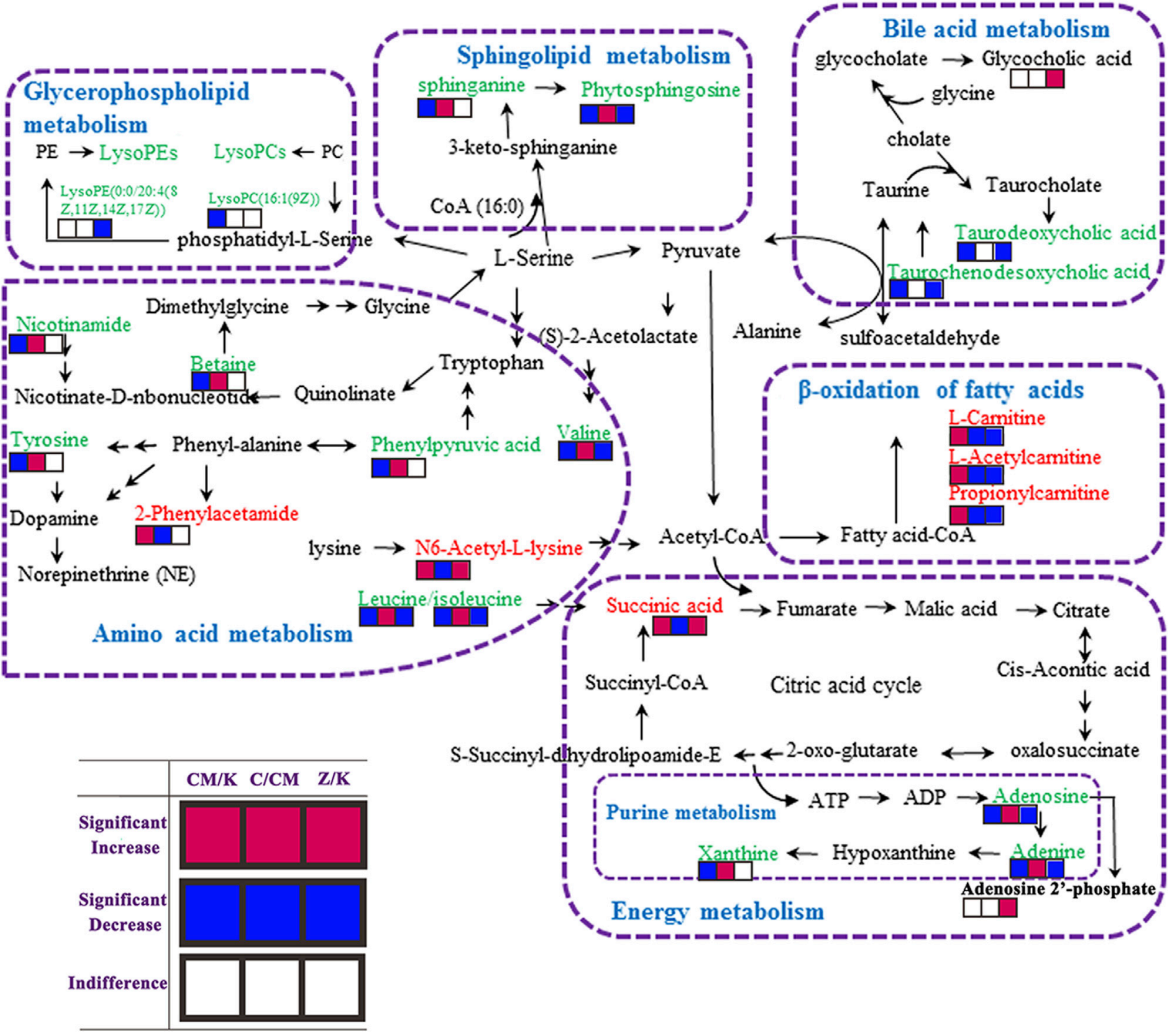
Metabolic pathways involved in the [CM] group versus the [K] group, the [CM] group versus the [C4], [C6] or [C7] group and the [Z4], [Z6] or [Z7] group versus the [K] group. Red squares represent up-regulated metabolites in the [CM] vs. [K], [C] vs. [CM] and [Z] vs [K] group; Blue squares represent down-regulated metabolites in the [CM] vs. [K], [C] vs. [CM] and [Z] vs. [K] group; And blank squares represent indistinctive metabolites in the [CM] vs. [K], [C] vs. [CM] and [Z] vs. [K] group. [CM] CUMS rats; [K] control rats; [C4], [C6], and [C7] CUMS rats given PBR (12.6, 50, 100 g/kg); [Z1], [Z6] and [Z7] control rats administered PBR (12.6, 50, 100 g/kg).

**FIGURE 7:**
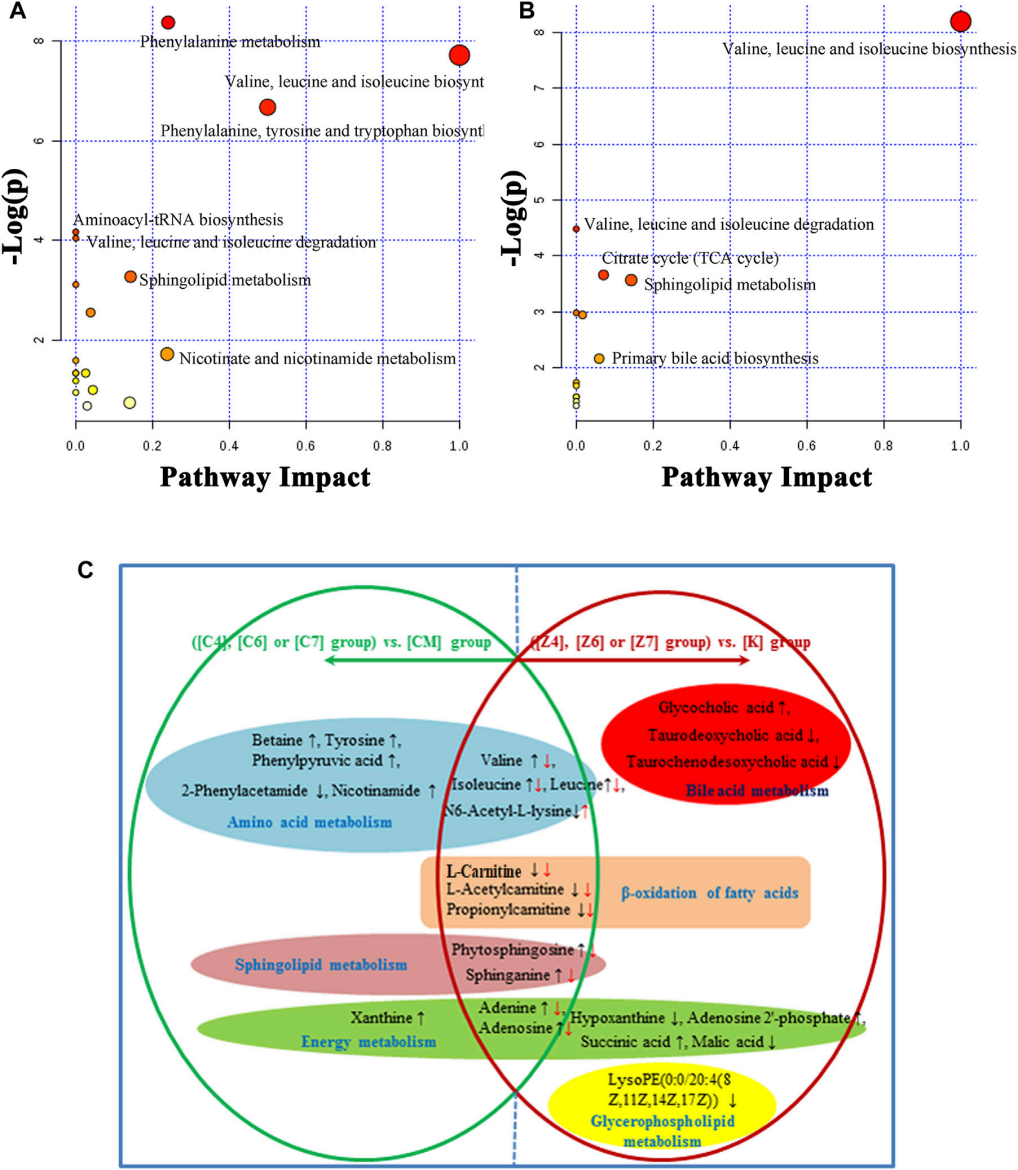
**(A)** Summary of pathways analysis of CUMS rats with PBR treatment with MetaboAnalyst. **(B)** Summary of pathways analysis of healthy rats with PBR administration with MetaboAnalyst. Each point represents one metabolic pathway; the size of dot and shades of color are positively correlated with the impact of the metabolic pathway. **(C)** Different metabolites and corresponding pathways in CUMS rats or healthy rats following PBR administration. “↑” and “↓” represent that the metabolite is up- or down-regulated in CUMS rats or healthy rats following PBR administration. [CM] CUMS rats; [K] control rats; [C4], [C6], and [C7] CUMS rats given PBR (12.6, 50, 100 g/kg); [Z1], [Z6] and [Z7] control rats administered PBR (12.6, 50, 100 g/kg).

The metabolic pathway was established based on related literature and the KEGG database to understand the correlation between these potential biomarkers. These metabolites were linked to amino acid metabolism, energy metabolism, β-oxidation of fatty acids, sphingolipid metabolism, glycerophospholipid metabolism, and bile acid metabolism (Figure 6). Next, differential metabolite pathway enrichment was performed using MetaboAnalyst software. Seven amino acid metabolism and sphingolipid metabolism pathways were obtained by impact value > 0.1 and -logP > 4 (Figure 7A), such as valine, leucine, and isoleucine biosynthesis, valine, leucine, and isoleucine degradation, aminoacyl-tRNA biosynthesis, phenylalanine metabolism, among others.

#### Differential Metabolic Profile in Control Rats

A significant separation was observed in the PCA score plots of the [K], [Z4], [Z6], and [Z7] groups (Figure 4B), suggesting that PBR could affect the metabolic profile of control rats. The goodness values of R^2^ Y = 0.929 and Q^2^ = 0.516 in the OPLS models in [Z7] versus [K] were computed, demonstrating that the models were reliable (Figure 4D). The contributions of different variables in [Z7] and [K] were extracted from the corresponding OPLS S-plots (Figure 4F).

As a result, 20 metabolites were significantly perturbed by PBR in normal rats (Table 6). Compared with the [K] group, leucine, isoleucine, valine, hypoxanthine, adenine, adenosine, malic acid, sphinganine, phytosphingosine, lysoPE [0:0/20:4 (8Z, 11Z, 14Z, 17Z)], l-carnitine, l-acetylcarnitine, propionylcarnitine, taurodeoxycholic acid, taurochenodesoxycholic acid, and ascorbic acid-2-sulfate decreased significantly in the [Z4], [Z6], and [Z7] groups (Figures 5B,C,E). In contrast, the levels of other metabolites, including N6-acetyl-l-lysine, succinic acid, adenosine 2′-phosphate, and glycocholic acid, increased significantly in the [Z4], [Z6], and [Z7] groups. The changes in metabolites were associated with amino acid metabolism, energy metabolism, sphingolipid metabolism, glycerolphospholipid metabolism, fatty acid β-oxidation, and bile acid metabolism (Figure 6). Similarly, five pathways in amino acid metabolism, energy metabolism, sphingolipid metabolism, and bile acid metabolism were considered to be the most pertinent in control rats given PBR, such as valine, leucine, and isoleucine biosynthesis; valine, leucine, and isoleucine degradation; and sphingolipid metabolism (Figure 7B).

**TABLE 6:** Identified differential metabolites in the liver of healthy rats.

No	Metabolites	T_R_ (min)	m/z	Ion	Z4/K	Z6/K	Z7/K	Metabolic pathway
1	Leucine^b^	3.16	132.1025	M + H	↓^a^	↓^a^	↓^a^	Amino acid metabolism
2	Isoleucine^b^	3.42	132.1025	M + H	↓^a^	↓^a^	↓^a^	Amino acid metabolism
3	Valine^b^	1.40	118.0864	M + H	↓^a^	↓^a^	↓^a^	Amino acid metabolism
4	N6-Acetyl-l-lysine	3.31	189.1232	M + H	↑	↑^a^	↑^a^	Lysine degradation
5	Hypoxanthine	4.39	137.0458	M + H	↓	↓^a^	↓^a^	Purine metabolism
6	Adenine	3.79	136.062	M + H	↓^a^	↓^a^	↓^a^	Purine metabolism
7	Adenosine	3.46	268.1036	M + H	↓^a^	↓^a^	↓^a^	Purine metabolism
8	Adenosine 2′-phosphate	1.78	348.0701	M + H		↑^a^	↑^a^	Purine metabolism
9	Succinic acid	2.99	117.0185	M-H		↑^a^	↑^a^	Citrate cycle
10	Malic acid	1.69	133.0135	M-H		↓^a^	↓^a^	Citrate cycle
11	Sphinganine	14.41	302.3058	M + H	↓^a^	↓^a^	↓^a^	sphingolipid metabolism
12	Phytosphingosine	11.73	318.3004	M + H	↓^a^	↓^a^	↓^a^	sphingolipid metabolism
13	LysoPE [0:0/20:4 (8Z,11Z,14Z,17Z)]	16.59	502.2918	M + H	↓	↓	↓^a^	Glycerophospholipid metabolism
14	l-Carnitine	1.39	162.1126	M + H		↓^a^	↓^a^	β-oxidation of fatty acid
15	l-Acetylcarnitine	2.36	204.1229	M + H			↓^a^	β-oxidation of fatty acid
16	Propionylcarnitine	4.45	218.1386	M + H	↓^a^	↓^a^	↓^a^	β-oxidation of fatty acid
17	Glycocholic acid	9.98	464.3021	M-H	↑	↑^a^	↑^a^	Bile acid metabolism
18	Taurodeoxycholic acid^b^	8.34	498.2897	M-H	↓	↓^a^	↓^a^	Bile acid metabolism
19	Taurochenodesoxycholic acid	10.27	498.2897	M-H	↓	↓^a^	↓^a^	Bile acid metabolism
20	Ascorbic acid-2-sulfate	1.82	254.9817	M + H			↓^a^	oxidative stress

Note: [K] control rats, [Z1] ∼ [Z7] control rats administered PBR (7 different dosages). ↑ shows up-regulated metabolite; ↓ shows down-regulated metabolite.

a Means a statistically significant difference at *p* < 0.05.

b Validated with standard. [Z4] group compared with [K] group, Z4/K; [Z6] group compared with [K] group, Z6/K; [Z7] group compared with [K] group, Z7/K.

### Metabolic Network Analysis of Metabolites in Both CUMS and Control Rats

Metabolites in both CUMS and control rats, six of them were returned to a healthy level after PBR treatment of CUMS-induced depression, including betaine, tyrosine, 2-phenylacetamide, phenylpyruvic acid, nicotinamide, and xanthine, while they were not significantly affected in control rats. This result indicated that these six metabolites were highly correlated with the pharmacological effects of PBR and were associated with amino acid metabolism, sphingolipid metabolism, and energy metabolism (Figure 7C).

In addition, another 11 metabolites showed the same regulative differential metabolites in both CUMS and control rats administered with PBR (*p* < 0.05, Figure 7C), including valine, isoleucine, leucine, N6-acetyl-l-lysine, phytosphingosine, sphinganine, adenine, adenosine, l-carnitine, l-acetylcarnitine, and propionylcarnitine, which were associated with amino acid metabolism, fatty acid β oxidation, sphingolipid metabolism, and energy metabolism (Figure 7C). Among these, 3-acyl carnitines (l-carnitine, l-acetylcarnitine, and propionyl carnitine) were decreased after PBR administration in both control and CUMS rats and could be significantly reversed to the level of the [K] group in the [C4] and [C6] groups. The other eight differential metabolites were reversed in both CUMS and control rats, indicating that the PBR might regulate the same sites under different body conditions, which caused it to present an opposite change tendency.

The rest of eight metabolites were significantly altered in control rats after PBR treatment, including glycocholic acid, taurodeoxycholic acid, taurochenodesoxycholic acid, hypoxanthine, adenosine 2′-phosphate, malic acid, succinic acid, and lysoPE (0:0/20:4 (8Z, 11Z, 14Z, 17Z)), which were associated with energy metabolism, glycerophospholipid metabolism, and bile acid metabolism (Figure 7C).

**FIGURE 8:**
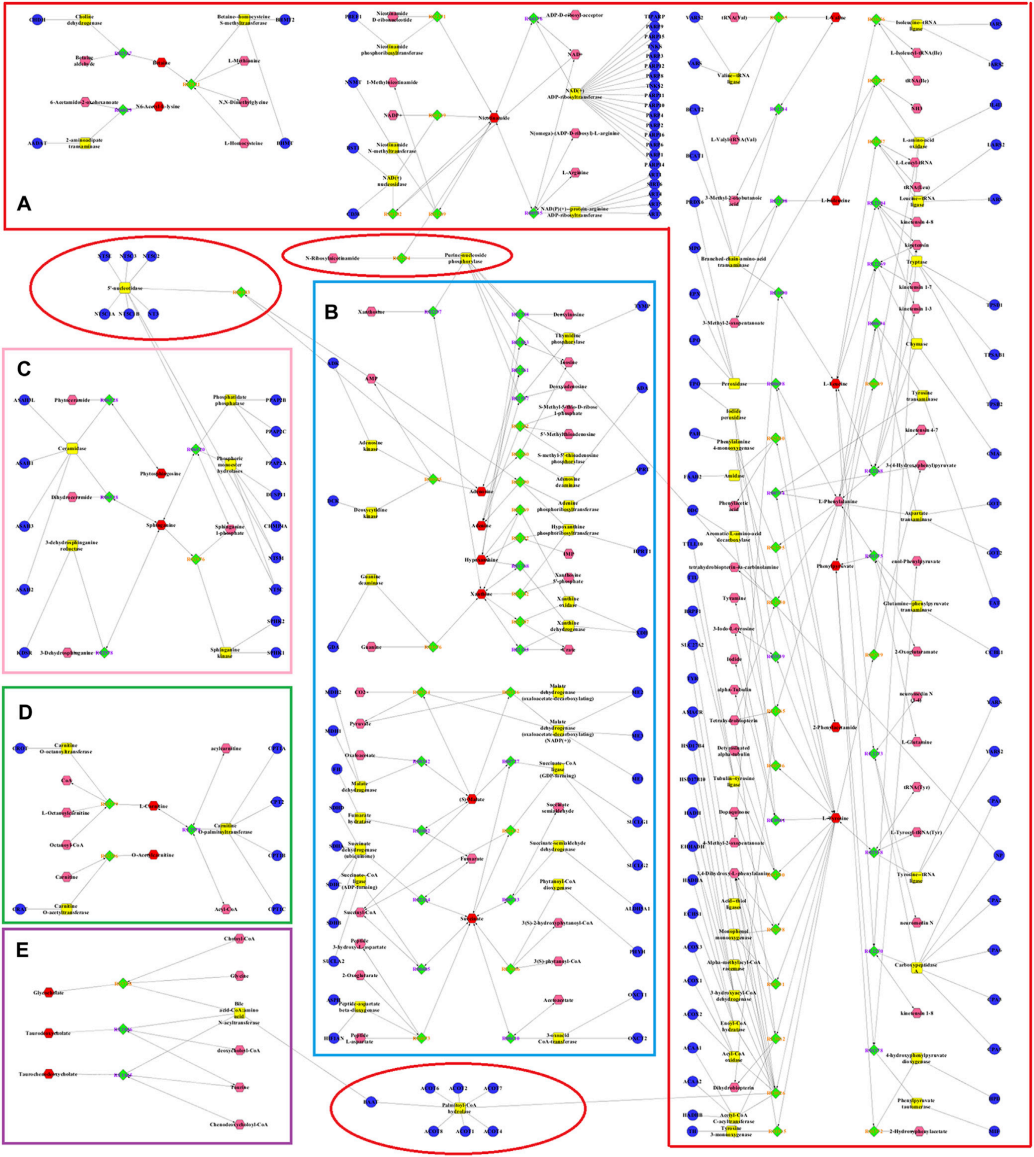
Metabolic networks involved in enzymes and genes were established based on the differential metabolites in both CUMS and healthy rats following PBR treatment. The metabolic networks of amino acid metabolism **(A)**, energy metabolism **(B)**, sphingolipid metabolism **(C)**, β-oxidation of fatty acid **(D)** and bile acid metabolism **(E)**.

The metabolic networks involved in some enzymes and genes were constructed using Cytoscape to understand better the potential biomarkers’ internal correlation in terms of enzyme or gene levels. The metabolic networks that were established based on the markedly differential metabolites are shown in Figure 8. Valine, leucine, isoleucine, and others involved in amino acid metabolism are shown in Figure 8A. Adenine, xanthine, malic acid, and others were also involved in energy metabolism (Figure 8B). As shown in Figures 8C–E, phytosphingosine, and sphinganine were involved in sphingolipid metabolism, l-carnitine and O-acetylcarnitine were involved in the β-oxidation of fatty acids, and glycocholate and taurodeoxycholate were involved in bile acid metabolism.

### Dose-Effect/Toxicity Relationship Analysis

#### Dose-Effect and Dose-Toxicity Metabolites Screening

**FIGURE 9:**
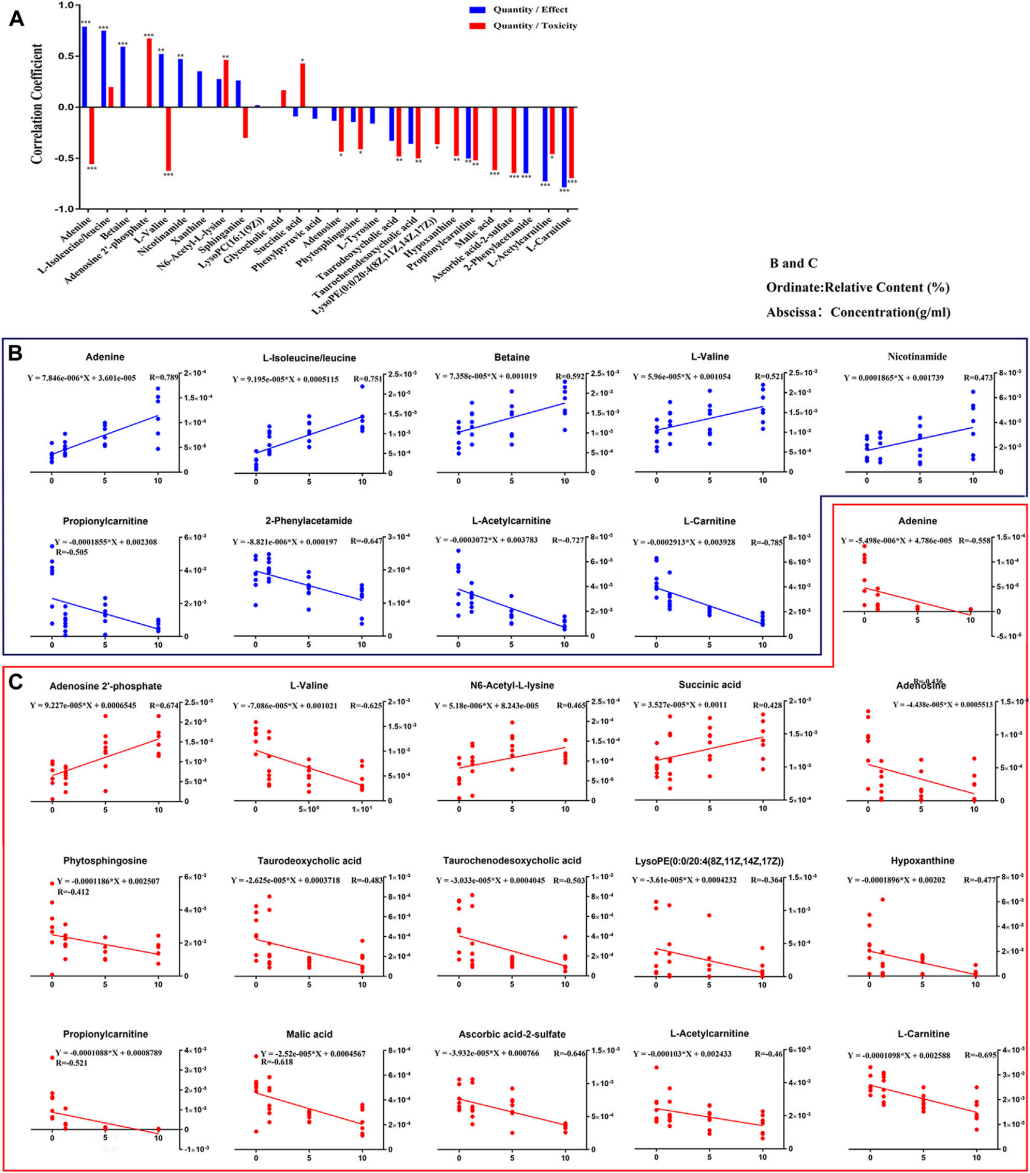
Relationships involved in Dose-Effect and Dose-Toxicity were established based on the differential metabolites in both CUMS and healthy rats following PBR treatment. Correlation of Quantity/Effect and Quantity/Toxicity **(A)**. Regression curve of Dose-Effect **(B)** and Dose-Toxicity **(C)**.

The relationship of dose-effect/toxicity was mainly to show the dose-dependence of metabolites by calculating the correlation between metabolite concentrations and dose. The correlations of dose-effect and dose-toxicity are shown in Figure 9A. Nine of the metabolites were significantly related to the drug effect (*p* < 0.05), including adenine, isoleucine/leucine, betaine, l-valine, nicotinamide, propionylcarnitine, 2-phenylacetamide, l-acetylcarnitine, and l-carnitine. Sixteen metabolites were significantly related to drug toxicity (*p* < 0.05), including adenine, adenosine-2′-phosphate, l-valine, N6-Acetyl-l-lysine, adenosine, phytosphingosine, taurodeoxycholic acid, lysoPE [0:0/20:4 (8Z, 11Z, 14Z, 17Z)], hypoxanthine, l-acetylcarnitine, malic acid, and ascorbic acid-2-sulfate. In Figure 9B, the regression curve of dose-effect and dose-toxicity showed the capability of evaluating the intensities of the relationship. Five pharmacodynamic metabolites, including isoleucine/leucine, l-valine, 2-phenylacetamide, l-acetylcarnitine, and l-carnitine, or five toxic metabolites including adenosine-2′-phosphate, l-valine, l-acetylcarnitine, malic acid, and ascorbic acid-2-sulfate were screened with R > 0.6.

#### Model Comparison

**FIGURE 10:**
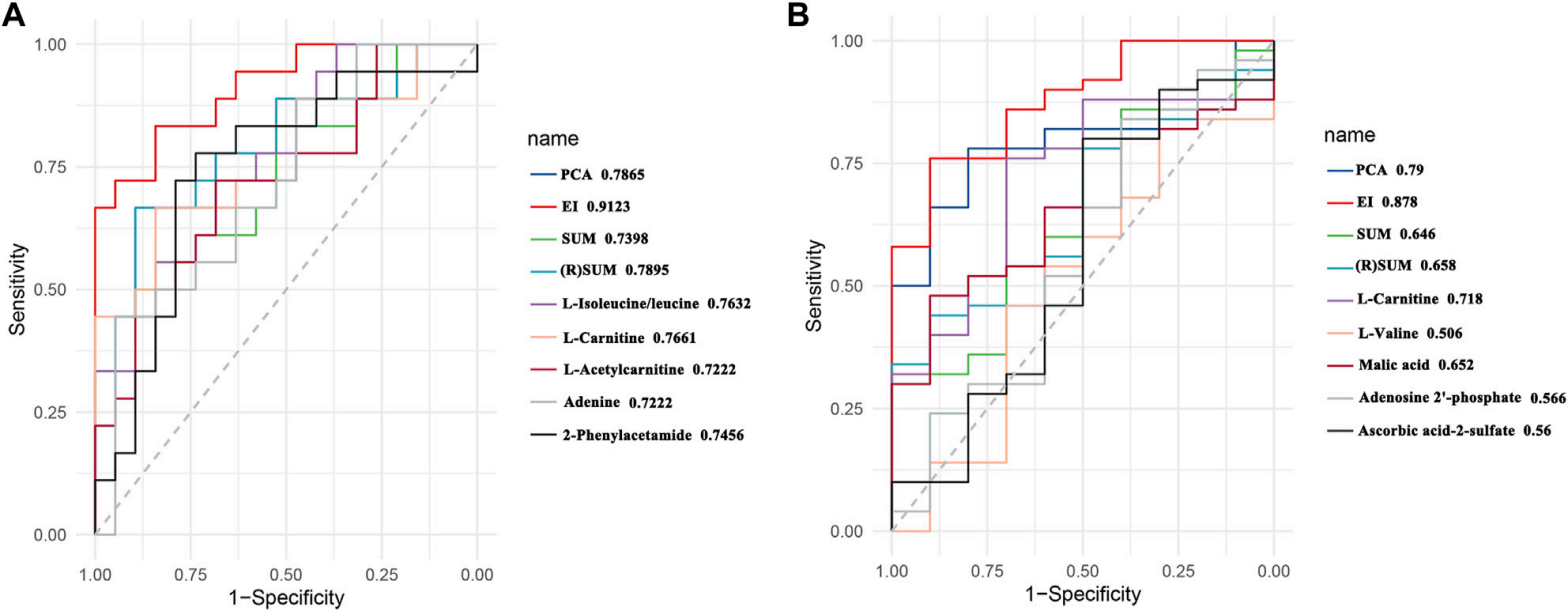
Receiver operating characteristic (ROC) curves of effect **(A)** and toxicity **(B)**.

To better evaluate the dose-effect/toxicity relationship of PBR, four computational methods including PCA analysis, EI analysis, the sum of metabolites (SUM), and (R)SUM were used to integrate the pharmacodynamic and toxic metabolites. The receiver operating characteristic curves (ROC) was applied to evaluate the computational methods. The evaluation results of the five pharmacodynamic metabolites and five toxic metabolites are shown in Figure 10 (ROC, 0.7222–0.7632 and 0.506–0.718), indicating that the metabolites have some ability to evaluate effect and toxicity. Furthermore, the four computational methods’ ROC was 0.7398–0.9123 and 0.658–0.878, suggesting that all computational methods could improve the evaluation ability of metabolites to different degrees. The improvement in EI analysis was the most significant, indicating that the EI analysis was suitable for integrating metabolites. Therefore, EI analysis was used to calculate the dose-effect/toxicity indices (DEI/DTI) and estimate the dose-effect/toxicity relationship.

#### Dose-Effect/Toxicity Indices

**FIGURE 11:**
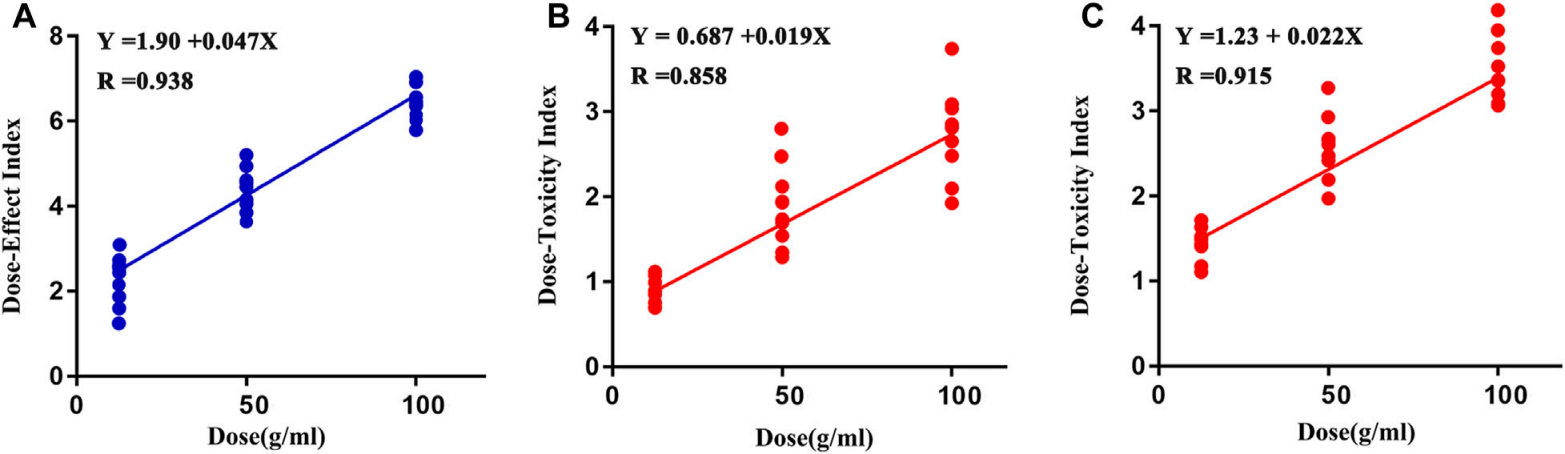
Regression curve of dose-effect/toxicity relationship in CUMS **(A, B)** and normal rats **(C)**.

The values of DEI and DTI in both CUMS and control rats following PBR treatment with C4, C6, and C7 dosage are shown in Figure 11. The DEI and DTI significantly increased with dosage augment. Regression models were used for the dose-effect/toxicity relationship assessment (Figure 11). The results showed that DEI and DTI exhibited a strong dose-effect/toxicity relationship evaluation (R > 0.85). The regression curve of the dose-effect/toxicity relationship in CUMS rats was y = 1.90 + 0.047x and y = 0.687 + 0.019 x. In normal rats, the regression curve of dose-toxicity was y = 1.23 + 0.22x. The results of probit regression analysis are shown in Supplementary Table S4, and the effective dose range for the CUMS rats was from 13.8 to 95.58 g/kg, the TC50 for CUMS and normal rats was 480 and 153 g/kg. Combined with the results of BRI and LCI, the dose range of effect and toxicity became larger in CUMS, and smaller in normal rats. This difference might be resulting from the inconformity of dosage groups in the metabonomics analysis.

## Discussion

Bupleuri Radix, a top grade herbal drug in Shennong’s Materia Medica, soothes the liver and relieves stagnation. This study focused on the effective and safe dose of PBR and its dose-effect/toxicity relationship. First, the BRI and LCI were obtained by integrated behavioral and liver indices and used to determine the dose range of effect and toxicity. The results showed that the effective dose range for CUMS rats was 12.6–163g (herb)/kg, the TD50 (median toxicity dose) for CUMS and normal rats were 388 and 207 g (herb)/kg, and the toxicological results showed that rats administrated with 100 g/kg PBR exhibited severe liver damage. After a comprehensive analysis, the use of PBR dose was determined to be 12.6–50 g (herb)/kg. Second, liver metabonomics was applied to gain insight into the related mechanisms, and the results showed that PBR could reverse amino acid metabolism, energy metabolism, sphingolipid metabolism, and β-oxidation of fatty acids based on liver metabolic profiles to produce an anti-depressant effect in a dose-dependent manner in CUMS rats. Extra two metabolic pathways, including glycerophospholipid metabolism and bile acid metabolism, were significantly perturbed in normal rats administered PBR. Finally, the dose-effect index (DEI) and dose toxicity index (DTI) were obtained by integrating the effects and toxic metabolites and were applied to precisely evaluate the dose-effect/toxicity relationship of PBR. The results showed that DEI and DTI had a remarkable ability to estimate the effect and toxicity. In addition, the DEI and DTI was used to determine the dose range of effect and toxicity, and it demonstrated high concordance with pre-experiment results. The CUMS possessed a higher toxicity tolerance dose of PBR, which was consistent with the theory of “You Gu Wu Yun” in TCM. “You Gu Wu Yun” theory suggested that the toxic herb would not produce toxicity in a corresponding pathological state; on the contrary, it would have a therapeutic effect (Tan et al., 2013).

In this study, behavioral research suggested that PBR had a positive anti-depressant effect on CUMS rats but no distinct impact on control rats. However, there were larger intra-group errors in behavioral tests, and liver function, especially in the sucrose preference test. This may be due to the individual differences in animals. Consequently, larger sample size is needed to obtain a meaningful statistical difference in the future. In addition, to comprehensively evaluate the efficacy and toxicity, the overall pharmacological potency was applied to evaluate the activity and toxicity of PBR, and the BRI and LCI, which were obtained by integrated behavioral tests and liver indices, were used to determine the dose range of effect and toxicity. The results showed that BRI and LCI could better evaluate the efficacy and toxicity and showed a dose-dependent effect.

CK-18F is considered a biomarker of cell death and has been used as a predictive indicator of drug-induced liver injury (DILI, Kakisaka et al., 2017). In this study, the content of CK-18F changed more in the liver than in the serum of CUMS and normal rats administered PBR. In control rats, the CK-18F level was increased in the Z5-Z7 group (25–100 g/kg) compared with the K group, indicating that the control rats showed a risk of DILI at 25–100 g/kg. However, in the CUMS rats, there was no significant change in CK-18F level at the dose of C5-C6 (25–50 g/kg), but a significant increase at the dose of C7 (100 g/kg), indicating that CUMS stress attenuated the risk of DILI at the medium dose of PBR (25–50 g/kg), which was consistent with the theory of “You Gu Wu Yun” in TCM. However, this study does not verify the toxic dose of PBR. The *in vivo* and *in vitro* experiments will be used to verify the findings of the study in the next step.

The metabolomic changes were performed using the metabolomics method in this study. In the protein precipitation method, the effects of precipitators (methanol, acetonitrile, acetonitrile-0.1% formic acid, methanol-0.1% formic acid) were compared. In the liquid chromatography conditions, the effects of different acids including formic acid, trifluoroacetic acid, and phosphoric acid, as well as the amount of acid (0.1, 0.2, and 0.3%) added into the mobile phase was compared and the chromatographic conditions of the mobile phase and gradient elution system were optimized to gain more information of metabolites. In addition, The QC samples were added in the process and observed tightly clustered in the result of PCA, suggesting that UHPLC- MS was a stable and reliable instrument in this population. The standards of metabolites were used to perform and ensure the accuracy of identification.

The perturbation of the amino acid neurotransmitter system plays an important role in the pathogenesis of depression (Ni et al., 2008). Amino acid metabolism is shown in Figure 8A. PBR mainly regulated branched-chain amino acids and affects synthetic norepinephrine (NE) in the treatment of depression. The delivery of branched-chain amino acids through the blood-brain-carrier system is closely related to the rate of 5-HT synthesis. In addition, branched-chain amino acids, especially leucine, play a major role in the differentiation of glutamate and glutamine in astrocytes, thereby maintaining the steady-state balance of brain nitrogen. It also affects the function of the central nervous system (Shimomura and Harris, 2006). More importantly, leucine and isoleucine can increase the expression of BDNF in hippocampal neurons, and BDNF dominates the signal transduction pathways associated with depression (Furukawa-Hibi et al., 2011). In this study, the changes in leucine, isoleucine, and valine were consistent with the results of serum metabonomics (Gao et al., 2017), suggesting that branched-chain amino acids, including leucine, isoleucine, and valine, were potential markers for PBR in the treatment of depression. Both l-tyrosine and phenylalanine are synthetic precursors of NE (Meyers, 2000), while phenylalanine metabolism produces phenylpyruvic acid and 2-phenylacetamide. A study (Wang, 2001) reported that elevated phenylalanine/tyrosine ratios could cause damage to the nervous system, leading to depression, mental development defects, and mental disorders. In this study, tyrosine and phenylpyruvic acid were significantly decreased, and 2-phenylacetamide was significantly increased in the liver of CUMS rats, suggesting that the perturbation of phenylalanine metabolism might cause NE synthesis deficiency, leading to the occurrence of depression.

N6-acetyl-l-lysine is an acetylated lysine. Acetylation of lysine is a reversible modification of the protein residue after translation, which has been considered as a novel regulatory factor of mitochondrial bioenergy in recent years, and the regulatory factor controls massive cellular life processes (Anderson and Hirschey, 2012; Thapa et al., 2017). The increase of N6-acetyl-l-lysine in the liver of CUMS rats indicated that stress might disturb healthy mitochondrial biological function. Betaine is an osmotic pressure molecule that accumulates in tissues, regulating cell volume (Schliess and Haussinger, 2002; Lang, 2007). It is also an important methyl donor so that homocysteine can methylate to methionine and plays a vital role in biological functions (Lever and Slow, 2010). Nicotinamide is involved in the tryptophan/kynurenine metabolic pathway. Additionally, both betaine and nicotinamide have a synergistic effect on synthetic anti-depressant drugs. The combination of betaine and s-adenosylmethionine in treating patients with mild to moderate depression is superior to s-adenosylmethionine alone. The combination of nicotinamide and tryptophan can significantly enhance the therapeutic effects by reducing the peripheral catabolism of tryptophan (Chouinard et al., 1977). In this study, the levels of amino acids and their metabolites were significantly decreased in CUMS rats. After the administration of PBR, these metabolites were significantly reversed to healthy, indicating that PBR produced anti-depressant effects by regulating amino acid neurotransmitter system metabolism. However, the branched-chain amino acids (leucine, isoleucine, and valine) were significantly reduced in the liver of control rats with PBR treatment, indicating that the amino acid transport was obstructed so that hepatic cells could not effectively absorb amino acids and ultimately cause liver injury. In this study, the levels of N6-acetyl-l-lysine were significantly increased in the liver of control rats with PBR treatment, indicating that the enhancement of lysine acetylation may disturb mitochondrial and cell functions and lead to liver cell damage.

The metabolic networks of energy metabolism involved in potential enzymes and genes are shown in Figure 8B. Energy is an indispensable factor in the survival of an organism. It has been reported that insufficient energy is closely related to depression. The conversion of adenine produces xanthine. Both xanthine and adenine are intermediate metabolites of adenosine, which play an important role in converting ATP and ADP (Su et al., 2014). In this study, the levels of xanthine, adenine, and adenosine were significantly decreased in the liver of CUMS rats, indicating that depression could weaken adenosine metabolism and reduce the function of energy conversion pathways in depressed patients. Similarly, malic acid and succinic acid are intermediates of the tricarboxylic acid (TCA) cycle. Their presence in the [CM] group indicated that the TCA cycle and energy metabolism in patients with depression was disturbed. After the administration of PBR, the reduced concentrations of xanthine, adenine, and adenosine in the liver were significantly reversed to healthy, indicating that PBR produced anti-depressant effects by regulating energy metabolism. However, the levels of hypoxanthine, adenine, and adenosine were significantly reduced, and adenosine 2′-phosphate was increased in the liver of control rats with PBR treatment, indicating that high doses of PBR may cause the adenosine metabolism to weaken and abnormal, and then cause the function of energy conversion pathways to attenuate, ultimately leading to hepatic cell damage due to insufficient energy supply for the survival of hepatic cells. Similarly, malic acid and succinic acid are intermediates in the TCA cycle, and their levels are abnormal in the liver of control rats with PBR treatment, indicating that high doses of PBR may lead to TCA weakening and abnormalities, leading to hepatic cell damage due to insufficient supply for normal survival processes of the hepatic cell.

Sphingolipid metabolism is shown in Figure 8C. Sphingolipids are an important component of meningeal lipids. An increased concentration of sphingolipids is closely related to depression (Dinoff et al., 2017). A study reported that sphingolipid levels in serum samples were significantly higher in depressed patients than in healthy individuals (Gracia-Garcia et al., 2011). In this study, the levels of phytosphingosine and sphinganine were significantly decreased in the livers of CUMS rats. After the administration of PBR, their levels in the liver of CUMS rats were significantly reversed to healthy, indicating that PBR produced anti-depressant effects by regulating sphingolipid metabolism. It has been reported that sphingomyelinase activation is a response to tumor necrosis factor-alpha (TNF-α) and other cytokines. Sphingomyelinase activation and C16-ceramide production are involved in TNF-α-induced hepatocyte apoptosis (Kolesnick et al., 1998). In addition, the dynamic balance of intracellular ceramide and sphingosine 1-phosphate (ceramide/S1P) may determine cell survival (Osawa et al., 2005), suggesting that sphingolipids are closely linked to the activity and survival of cells. In this study, the PBR produced disturbances to endogenous metabolites of hepatic sphingolipids in control rats administered with PBR, including sphinganine and phytosphingosine, suggesting that high-dose PBR might cause liver damage by altering hepatocyte survival and membrane structure.

The metabolic networks of β-oxidation of fatty acids are shown in Figure 8D β-oxidation of fatty acids is an important pathway for fatty acid decomposition and energy production, and its abnormality can lead to dysfunction of the nervous system. Acyl carnitines are long-chain acyl fatty acid esters of carnitine. They can carry long-chain fatty acids from the cytoplasm into the mitochondria, allowing long-chain fatty acids to oxidize in the mitochondria to produce energy. However, this transportation is dependent on carnitine (Malaguarnera et al., 2011; Ren et al., 2013). In this study, the levels of l-carnitine, l-acetylcarnitine, and propionylcarnitine were significantly increased in the liver of CUMS rats, indicating that the transportation of long-chain fatty acids into the mitochondria was disturbed, thereby interfering with the energy production of the long-chain fatty acid oxidation process. After the administration of PBR, the concentrations of l-carnitine, l-acetylcarnitine, and propionylcarnitine in D4 and D6 were significantly reversed to healthy, indicating that PBR produced an anti-depressant effect by regulating β-oxidation of fatty acids. Another study (Devaux, 1991) reported that acetyl-l-carnitine treatment improved liver function and quality of life in patients with mild hepatic encephalopathy. Therefore, in normal rats, the levels of acyl-carnitine were significantly decreased in the liver of control rats administered with PBR, indicating that PBR may alter acyl-carnitine to cause mitochondrial β-oxidation of fatty acid dysfunction, resulting in energy deficiency and eventually leading to liver damage.

The glycerophospholipid metabolism was only significantly changed in the livers of control rats administered with PBR. Lysophosphatidylethanolamine (LysoPEs) is produced by the metabolism of phosphatidylethanolamine (PE). Both PE and sphingolipids are the main components of cell membrane phospholipids and are distributed asymmetrically in the plasma membrane. Most PEs are embedded in the inner membrane of the cell membrane and constitute the membrane phospholipid bilayer (Jaeschke et al., 2002). The abnormality of LysoPEs in control rats administered with PBR suggested that PEs were perturbed, further affecting the plasma membrane structure and permeability. In this study, LysoPE [0:0/20:4 (8Z, 11Z, 14Z, 17Z)] was significantly decreased in the livers of control rats administered with PBR, indicating that PBR might cause liver damage by changing the structural integrity and permeability of the plasma membrane in normal rats. Bile acid metabolism is shown in Figure 8E. Bile acid metabolism was also significantly changed in control rats administered with PBR. Bile acids are sensitive indicators of liver and liver damage. Abnormally elevated bile acids, such as cholestasis, can cause the accumulation of toxic bile acids in the liver, leading to pathophysiological effects, including mitochondrial dysfunction and overproduction of reactive oxygen and nitrogen (Palmeira and Rolo, 2004; Tan et al., 2007). More importantly, slight liver damage can cause bile acid perturbation in the serum and liver (Yamazaki et al., 2013). Various liver diseases, such as non-alcoholic fatty liver disease and drug-induced liver injury, can increase intrahepatic bile acid levels (Lake et al., 2013). In this study, glycocholic acid was significantly elevated in the liver, which was consistent with the results of serum metabonomics of control rats administered with PBR, indicating that high-dose PBR might accumulate intrahepatic bile acid and cause liver injury.

In this study, 8,671 metabolites were measured, and 409 metabolites were identified in the sample. The metabolites were mainly high polarity compounds including amino acids, sphingolipids, bile acids, etc. While the less polarity compounds such as fatty acids and lipid metabolites were difficult to be detected. The GC-MS serum metabolomics was performed and the fatty acids were detected in our previous study (Gao, et al., 2014). The results showed that depression was associated with amino acid metabolism and energy metabolism, which were consistent with the results of this study. Therefore, the targeted metabolomics was applied to quantify the metabolites of amino acid metabolism and energy metabolism in follow-up studies.

The dose-effect/toxicity relationship is the essence of the clinical use of TCM. Because of the multi-component and multi-target characteristics of TCM (Wang et al., 2010), a comprehensive evaluation of the dose-effect/toxicity relationship is important to allow its application in modern medical practice. Although some convenient and effective evaluation indices have been used in previous dose-effect/toxicity studies, including body weight, blood pressure, blood glucose, transaminase, platelets, and cell number, there are still many disease effect indices that lack quantification, and depression is a major disease. With the development of studies regarding the essence of TCM syndrome based on metabonomics, the importance of metabonomics for evaluating overall effects was gradually being recognized (Wang et al., 2010). After administration, the endogenous small molecules shifted in the same direction as the dose increased. Metabolites representing the organic state were screened and integrated into a dose-effect/toxicity relationship analysis. A significant dose-effect/toxicity relationship was observed with a high dose-dependence. The results showed that the comprehensive index was better for evaluating the dose-effect/toxicity relationship, which is consistent with the fact that it may be caused by the multi-component and multi-target characteristics of the TCM. In this study, four-function models were compared for metabolite integration, and the results showed that the different models might influence the evaluation characteristics of the metabolites. Therefore, exploring a more suitable functional model would help construct a metabolic evaluation system for depression. However, it is necessary to verify effect/toxicity biomarkers of PBR. The vitro cell experiments would be used to verify the specificity and accuracy of toxicity biomarkers in follow-up studies.

## Conclusions

The current study demonstrates that the effective dose range and median toxicity dose of PBR for CUMS rats are 12.6–50 g (herb)/kg and 388 g (herb)/kg, and PBR produces anti-depressant effects by reversing amino acid metabolism, energy metabolism, sphingolipid metabolism, and β-oxidation of fatty acids in CUMS rats. In control rats, the median toxicity dose of PBR is 207 g (herb)/kg, and extra two metabolic pathways including glycerophospholipid metabolism and bile acid metabolism are significantly perturbed after administration with PBR. Moreover, the comprehensive metabolic indexes including DEI and DTI have a remarkable ability to predict effect and toxicity which needs further follow-up validation. Furthermore, the CUMS rats possessed a higher toxicity tolerance dose of PBR, which was consistent with the theory of “You Gu Wu Yun”. These results indicate that the metabonomics techniques combined with correlation analysis could be used to discover indicators for comprehensive evaluations of efficacy and toxicity.

## Data Availability

The original contributions presented in the study are included in the article/Supplementary Material, further inquiries can be directed to the corresponding authors.

## Glossary

ALP: alkaline phosphatase
ALT: alanine aminotransferase
AST: aspartate aminotransferase
BRI: Behavioral Regulation Index
ceramide/S1P: ceramide and sphingosine 1-phosphate
CK-18F: cytokeratin-18 fragment
CUMS: chronic unpredictable mild stress
DEI: dose-effect index
DILI: drug-induced liver injury
DTI: dose toxicity index
EI: effect index
LCI: Liver Composite Index
LC-MS: the liquid chromatography with mass spectrometry
LysoPEs: Lysophosphatidylethanolamine
NE: norepinephrine
OPLS: orthogonal partial least squares
PBR: lower polar fraction of Bupleuri Radix
PCA: principal component analysis
PDA: Photodiode Array
PE: phosphatidylethanolamine
PLS-DA: partial least-squares discriminant analysis
QAMS: quantitative analysis of multiple components by a single marker
QC: Quality control
ROC: receiver operating characteristic
TBIL: total bilirubin
TCA: tricarboxylic acid
TCM: traditional Chinese medicine
TD50: median toxicity dose
TNF-α: tumor necrosis factor-alpha
TIC: total ions chromatograph
UHPLC: ultra-performance liquid chromatography system
