# Supplementary material for: Dose-Effect/Toxicity of Bupleuri Radix on Chronic Unpredictable Mild Stress and Normal Rats Based on Liver Metabolomics
Source: Front Pharmacol. 2021 Sep 7;12:627451. doi: 10.3389/fphar.2021.627451 (PMC8452938; doi:10.3389/fphar.2021.627451)
Supplement: Supplementary file 1 [file DataSheet1.docx]

**Dose-Effect/Toxicity of Bupleuri Radix on Chronic Unpredictable Mild Stress and Normal Rats based on liver metabolomics**

Peng Wang ^a, c#^, Xiaoxia Gao^a, b, #*^, Meili Liang^a,^ ^c^, Yuan Fang^a^, Jinping Jia^d^ , Junsheng Tian^a, b^, Xiang Zhang^a, e^, Xuemei Qin^a, b, *^

^a^ Modern Research Center for Traditional Chinese Medicine, Shanxi University, Taiyuan 030006, China

^b^ Key Laboratory of Chemical Biology and Molecular Engineering of Ministry Education of Shanxi University, Taiyuan 030006, China

^c^ College of Chemistry and Chemical Engineering, Shanxi University, Taiyuan 030006, China

^d^ Scientific Instrument Center, Shanxi University, Taiyuan 030006, China

^e^ University Of Louisville, Louisville, 40292, USA

**AUTHOR INFORMATION**

**First Author**

^#^Peng Wang and Xiaoxia Gao contributed equally.

**Corresponding Author**

^*^ Xiaoxia Gao, Phone: 86-351-7019297, Fax: 86-351-7011202, E-mail: [gaoxiaoxia@sxu.edu.cn;](mailto:gaoxiaoxia@sxu.edu.cn;)

^*^ Xuemei Qin, Phone: 86-351-7011501, Fax: 86-351-7011202, E-mail: [qinxm@sxu.edu.cn;](mailto:qinxm@sxu.edu.cn;)

**Author Contributions**

^△^These authors contributed equally to this work P.W., X.X.G., M.L.L., Y.F., conceived and designed the experiments; P.W. and X.X.G wrote the paper; M.L.L., Y.F., J.P.J performed the experiments; P.W., M.L.L., Y.F. analyzed the data; J.S.T., Z.Y.L., X.M.Q. design of the study and writing the protocol; X.Z. helpful revision on the text and grammar. All authors have given approval to the final version of the manuscript.


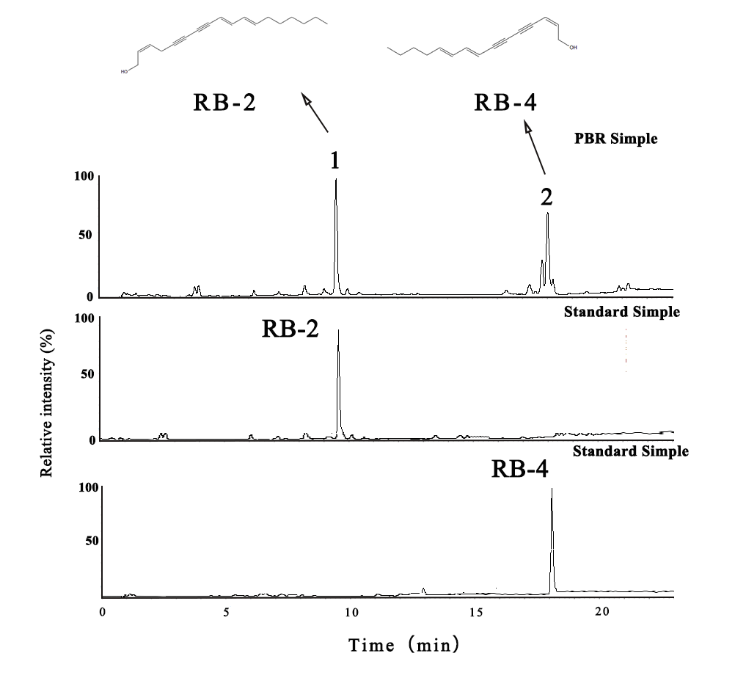


**Figure S1. Representative UPLC chromatograms of the RB-2 and RB-4 in PBR**

The linear regression equation of RB-2 and RB-4 was Y = 16450X-1538. 4（R=0.9992）and Y = 28 963X -3135. 1（R=0.9993）in our previous study (Zhang et al., 2017), the area is vertical coordinate (Y) and the corresponding content is horizontal coordinate (X). The contents of the RB-2 and RB-4 in PBR was 3.62mg/g and 1.88mg/g.


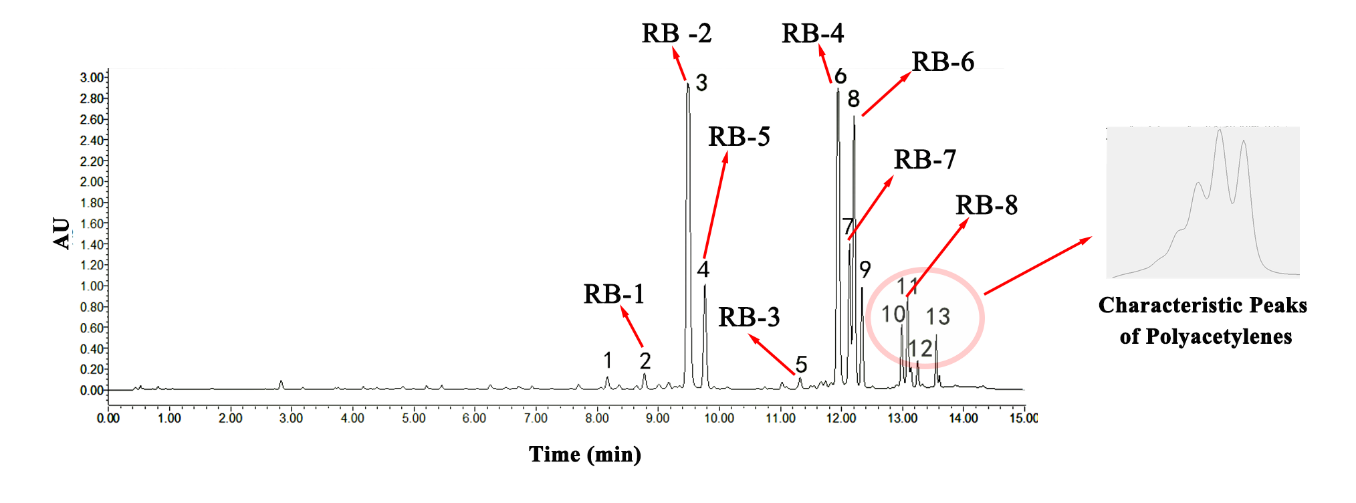


**Figure S2. Chromatograms of the Polyalkynes in PBR**


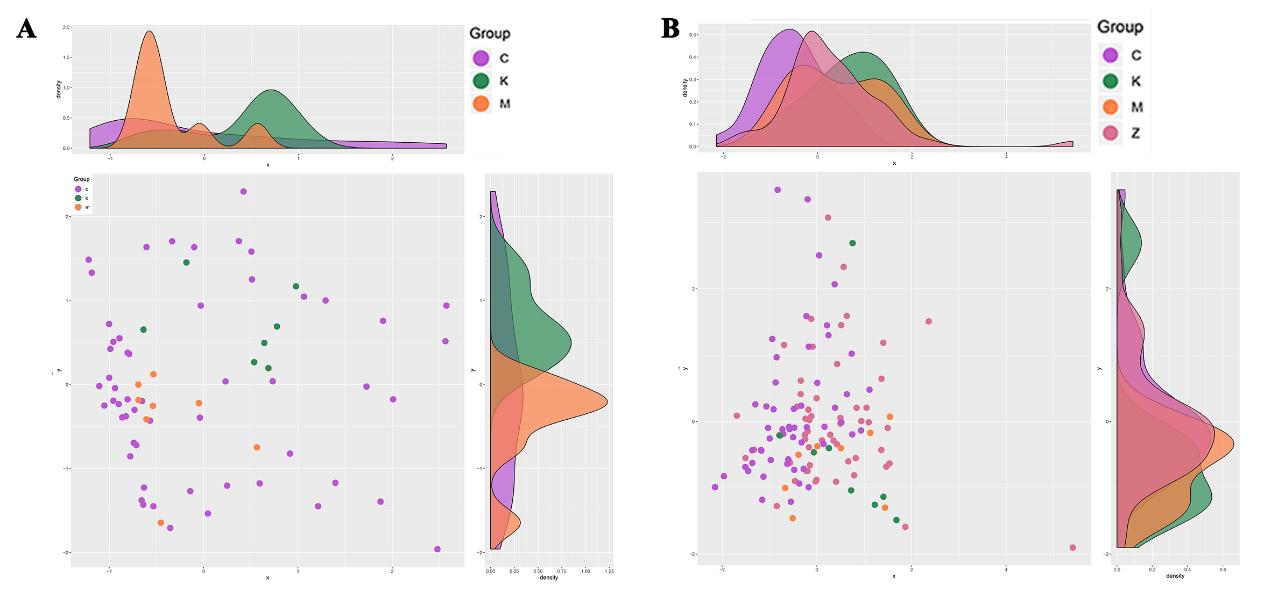


**Figure S3.** The principal components analysis (PCA) of BRI and LCI.


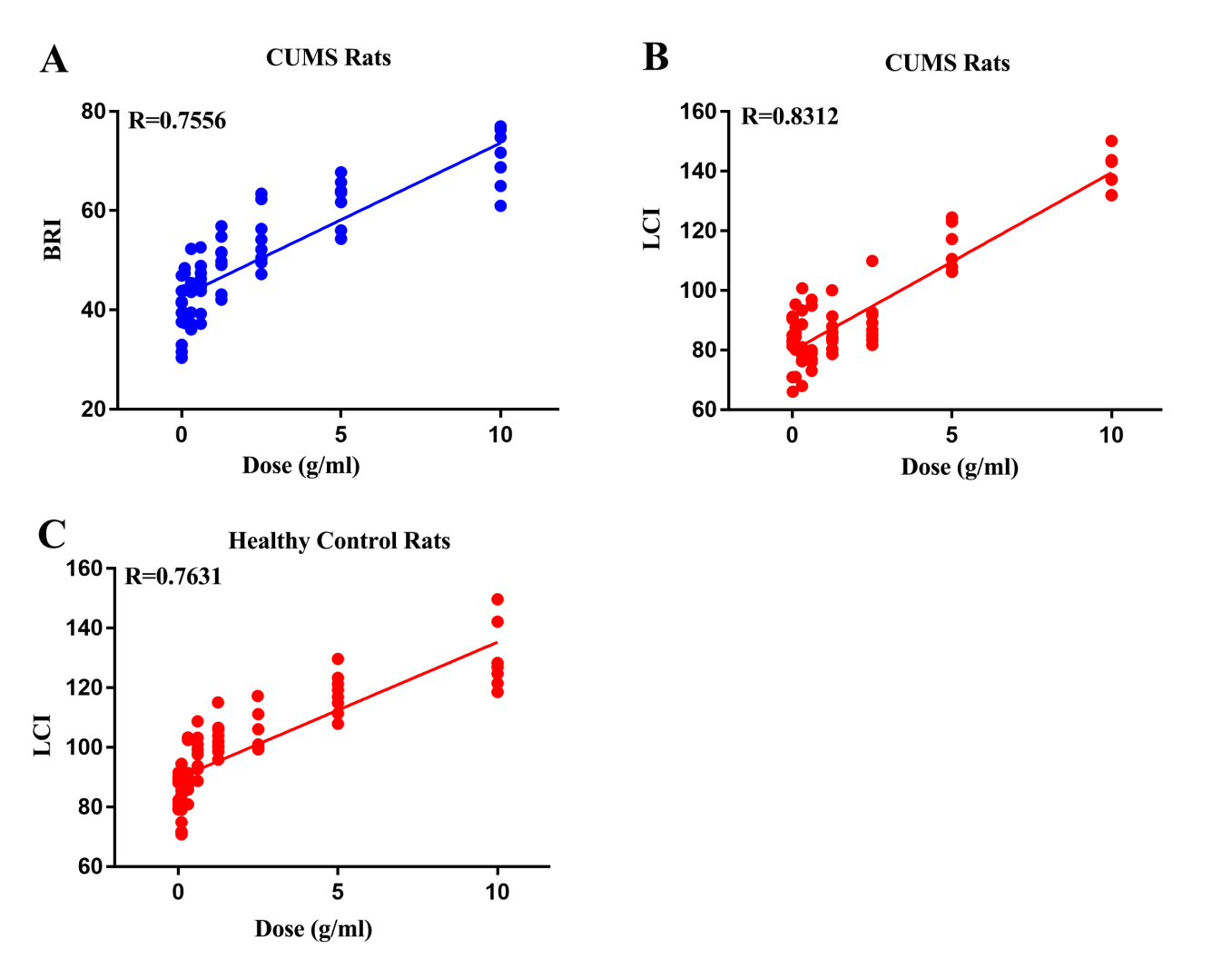


**Figure S4.** The regression curve of dose-effect/toxicity relationship in CUMS (A, B) and normal rats (C).


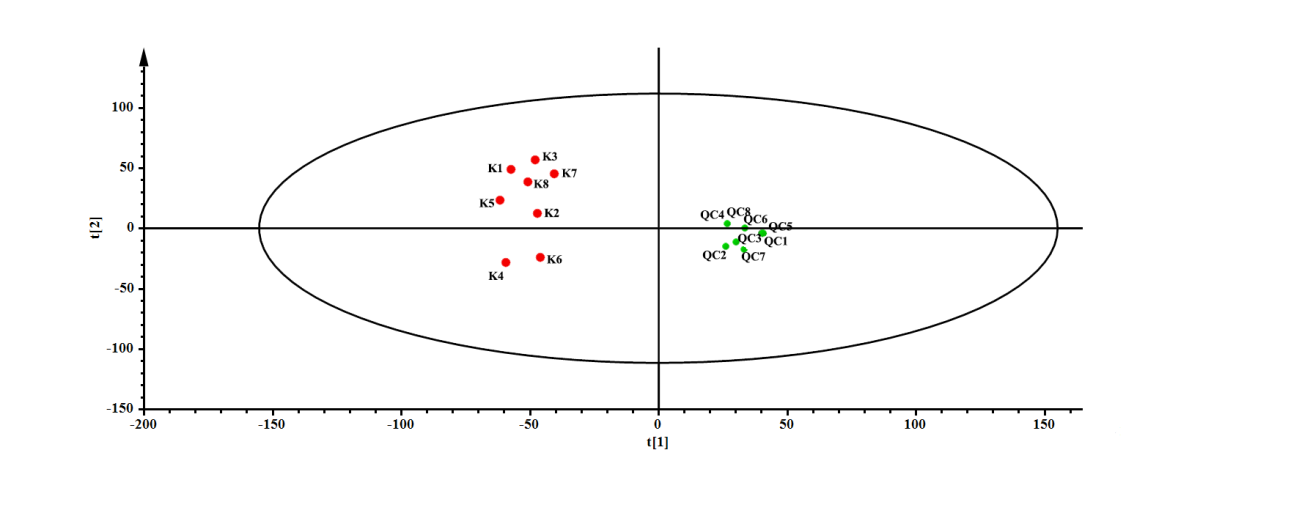


**Figure S5.** The principal components analysis (PCA) of the QC sample.

Table S1. List of stimulant factors of CUMS rats

| Stimulating Factor | Description |
| --- | --- |
| Water deprivation | Deprivation of drinking water for 24 hours |
| Thermal stimulation | Placing in an electric oven at 45℃ for 10 min. |
| Ultrasound stimulation | Stimulating with 60 dB ultrasound for 3 h. |
| Tail clamping | The rats were fixed and exposed to the tail and clamped 1 cm from the root of the tail for 2 min. |
| Constraint | Bounding to the binding plate, so that the rats’ limbs could not move freely and stimulated for 3 h. |
| Foot shocking | The rats were placed in a plantar electric shock box with a voltage of 36 V. The rats were subjected to electric shock every 10 s for 2 s for a total of 10 times. |
| Food deprivation | Deprivation of feed for 24 hours. |
| Day-night reversal | 24 h illumination. |
| Ice water swimming | The rats were placed in a plexiglass tank containing 4℃ ice water (20 cm, high 50 cm in diameter). The water depth was about 20 cm, when the rat tail touched the bottom of the cylinder. After swimming for 5 min, the rats were fished out, the towels dried and placed in a warm box to avoid spasms. The rats were returned to cage after all the signs were stable. |

**Table 2. Behavior on CUMS and healthy rats administration with PBR（X ± SD，n=8）**

| **Group** | **Weight** | | **Rearings** | | **Crossings** | | **Sucrose preference**  **value** | |
| --- | --- | --- | --- | --- | --- | --- | --- | --- |
|  | 0 day | 21 day | 0 day | 21 day | 0 day | 21 day | 0 day | 21 day |
| C1 | 269.4 ± 8.7 | 358.8 ± 6.1^**^ | 7.2 ± 3.3 | 3.6 ± 0.6 ^###^ | 44.0 ± 6.3 | 27.1 ± 4.8^#^ | 89.2 ± 12.8 | 49.0 ± 12.5^##^ |
| C2 | 265.5 ± 4.7 | 339.3 ± 6.2^*^ | 9.2 ± 4.9 | 3.5 ± 0.9 ^###^ | 22.5 ± 5.6# | 11.6 ± 2.5^##^ | 80.7 ± 20.0 | 67.0 ± 15.6 |
| C3 | 261.1 ± 6.0 | 345.7 ± 8.8^**^ | 11.5 ± 2.3 | 5.2 ± 0.8 ^##^ | 39.0 ± 21.3 | 32.2 ± 4.2^**^ | 65.6 ± 19.2 | 45.2 ± 8.6^##^ |
| C4 | 261.7 ± 6.0 | 347.9 ± 8.2^**^ | 11.3 ± 4.3 | 6.9 ± 1.4 ^*#^ | 41.5 ± 16.7 | 30.9 ± 4.6^**^ | 63.7 ± 14.5 | 33.2 ± 13.1^**##^ |
| C5 | 262.5 ± 1.2 | 337.4 ± 6.5^*^ | 8.3 ± 5.9 | 4.9 ± 1.4 ^###^ | 47.7 ± 16.0 | 30.0 ± 7.4^**^ | 84.6 ± 12.1 | 58.1 ± 19.1^#^ |
| C6 | 263.2 ± 3.4 | 337.8 ± 3.9^*^ | 11.3 ± 3.8 | 9.1 ± 2.6 ^***^ | 43.7 ± 14.2 | 49.0 ± 11.0^***^ | 73.9 ± 19.9 | 82.6 ± 9.0^*^ |
| C7 | 267.3 ± 13.8 | 314.1 ± 10.5^###^ | 9.0 ± 3.8 | 8.2 ± 1.8 ^***^ | 57.5 ± 15.2 | 56.4 ± 11.6^***^ | 69.3 ± 19.8 | 54.5 ± 15.6^#^ |
| Z1 | 261.8 ± 10.8 | 352.0 ± 8.6^***^ | 12.2 ± 4.6 | 14.6 ± 3.7 | 56.8 ± 11.3 | 43.5 ± 8.4 | 65.8 ± 13.7 | 75.8 ± 15.4 |
| Z2 | 261.8 ± 8.1 | 357.3 ± 5.3^***^ | 14.2 ± 4.1 | 18.5 ± 6.9 | 64.4 ± 16.6 | 56.2 ± 12.5 | 60.9 ± 16.5 | 77.8 ± 19.1 |
| Z3 | 254.9 ±5.6 | 358.4 ± 2.3^***^ | 7.5 ± 5.2 | 9.2 ± 4.8 | 59.7± 11.6 | 44.9 ± 12.1 | 77.0 ± 12.6 | 91.0 ± 18.2 |
| Z4 | 259.3 ±7.2 | 352.0 ± 3.4^***^ | 12.3 ± 2.6 | 13.9 ± 5.4 | 56.6 ± 14.6 | 39.0 ± 12.6 | 69.3 ± 15.9 | 79.6 ± 13.7 |
| Z5 | 251.5 ±6.6 | 353.6 ± 4.3 | 8.3 ± 7.8 | 11.9 ± 3.4 | 67.7 ± 14.1 | 50.8 ± 9.5 | 82.4 ± 11.5 | 93.2 ± 14.9 |
| Z6 | 253.6 ±4.4 | 348.8 ± 2.6^***^ | 12.3 ± 3.7 | 13.1 ± 7.6 | 63.9 ± 16.9 | 52.2 ± 12.6 | 79.1 ± 17.8 | 86.9 ± 19.4 |
| Z7 | 268.2 ±9.1 | 354.3 ± 7.3^***^ | 9.0 ± 2.9 | 11.2 ± 4.9 | 58.5 ± 13.3 | 46.5 ± 6.44 | 83.1 ± 11.9 | 94.5 ± 16.9 |
| CM | 263.0 ± 8.6 | 323.1 ± 4.0^###^ | 7.3 ± 4.5 | 3.7 ± 0.7 ^###^ | 45.8 ± 22.5 | 18.6 ± 4.2^##^ | 85.2 ± 11.9 | 66.4 ± 17.0^#^ |
| CY | 267.8 ± 13 | 341.3 ± 9.7^**^ | 7.8 ± 3.5 | 3.0 ± 1.0 ^###^ | 55.0 ± 14.1 | 24.0 ± 7.2^**^ | 66.3 ± 12.2 | 74.8 ± 14.2^*^ |
| K | 263.0 ± 7.7 | 351.5 ± 4.7^***^ | 10.7 ± 7.3 | 11.6 ± 1.6 ^**^ | 62.7 ± 19.7 | 45.8 ± 6.1^***^ | 78.7 ± 18.6 | 81.2 ± 12.1^*^ |

Note：#, compared with the K group (#p<0.05, ##P<0.01, ###P<0.001); *, compared with the M group (*P<0.05, **P<0.01, ***P<0.001)

**Table 3. The stability of UHPLC -Q Exactive Orbitrap -MS method using QC sample.**

| **NO.** | **T_R_(min)** | **RSD (%)** | **m/z** | **RSD (%)** | **ion** |
| --- | --- | --- | --- | --- | --- |
| 1 | 1.37 | 0.9432 | 118.0865 | 6.85×10^-5^ | M+H |
| 2 | 3.55 | 3.8518 | 132.1025 | 4.74×10^-5^ | M+H |
| 3 | 3.27 | 2.8801 | 182.0813 | 5.49×10^-5^ | M+H |
| 4 | 2.76 | 2.4200 | 153.0407 | 5.18×10^-5^ | M+H |
| 5 | 4.49 | 2.5104 | 218.1387 | 5.41×10^-5^ | M+H |
| 6 | 14.36 | 0.5928 | 302.3055 | 6.00×10^-5^ | M+H |
| 7 | 11.76 | 0.5904 | 318.3003 | 4.10×10^-5^ | M+H |
| 8 | 15.86 | 0.2238 | 494.3243 | 5.13×10^-5^ | M+H |
| 9 | 8.43 | 0.3453 | 500.3014 | 9.6610^-5^ | M+H |

**Table 4. The samples number** **of effecty and toxicity on CUMS and normal rats administration with PBR**

| **Dose**  **(g/kg)** | **Effecty** | | **Toxicity** | | | |
| --- | --- | --- | --- | --- | --- | --- |
|  | **CUMS rats** | | **CUMS rats** | | **Normal rats** | |
|  | **Total simples** | **positive samples** | **Total simples** | **positive samples** | **Total simples** | **positive samples** |
| 0 | 8 | 0 | 8 | 3 | 8 | 0 |
| 12.5 | 8 | 3 | 8 | 0 | 8 | 0 |
| 50 | 8 | 7 | 8 | 1 | 8 | 2 |
| 100 | 8 | 7 | 8 | 2 | 8 | 2 |

The obtained effective dose range for the CUMS rats was from 13.8 g/kg to 95.58g/kg (IC50 – IC95) with the regression function PROBIT(*P*)=-3.333+2.41$x$, and the TC50 for CUMS and normal rats was 480 g/kg and 153 g/kg with the regression function PROBIT(*P*)=-0.717+0.015$x$ and PROBIT(*P*)=-1.473+0.096$x$.
